# Tiny Rare-Earth Fluoride Nanoparticles Activate Tumour Cell Growth via Electrical Polar Interactions

**DOI:** 10.1186/s11671-018-2775-z

**Published:** 2018-11-21

**Authors:** Vadim V. Semashko, Maksim S. Pudovkin, Alkiviadis-Constantinos Cefalas, Pavel V. Zelenikhin, Vassilios E. Gavriil, Alexei S. Nizamutdinov, Zoe Kollia, Angelo Ferraro, Evangelia Sarantopoulou

**Affiliations:** 1 National Hellenic Research Foundation, Theoretical and Physical Chemistry Institute, 48 Vassileos Constantinou Avenue, 11635 Athens, Greece; 2 Institute of Physics, Kazan Federal University, 18 Kremljovskaja str, Kazan, 420008 Russia; 3 Department of Microbiology, Kazan Federal University, 18 Kremljovskaja str, Kazan, 420008 Russia

**Keywords:** Physics of cancer, Tumorigenesis, Cancer and nanoparticles, Mechanotransducers, Mechanosensors, Integrins, EGFR, Nanotechnology, Biosurfaces, Atomic force microscopy, Electrical dipole interactions

## Abstract

Localised extracellular interactions between nanoparticles and transmembrane signal receptors may well activate cancer cell growth. Herein, tiny LaF_3_ and PrF_3_ nanoparticles in DMEM+FBS suspensions stimulated tumour cell growth in three different human cell lines (A549, SW837 and MCF7). Size distribution of nanoparticles, activation of AKT and ERK signalling pathways and viability tests pointed to mechanical stimulation of ligand adhesion binding sites of integrins and EGFR via a synergistic action of an ensemble of tiny size nanoparticles (< 10 nm). While tiny size nanoparticles may be well associated with the activation of EGFR, integrin interplay with nanoparticles remains a multifaceted issue. A theoretical motif shows that, within the requisite pN force scale, each ligand adhesion binding site can be activated by a tiny size dielectric nanoparticle via electrical dipole interaction. The size of the active nanoparticle stayed specified by the amount of the surface charges on the ligand adhesion binding site and the nanoparticle, and also by the separating distance between them. The polar component of the electrical dipole force remained inversely proportional to the second power of nanoparticle’s size, evincing that only tiny size dielectric nanoparticles might stimulate cancer cell growth via electrical dipole interactions. The work contributes towards recognising different cytoskeletal stressing modes of cancer cells.

**Electronic supplementary material:**

The online version of this article (10.1186/s11671-018-2775-z) contains supplementary material, which is available to authorized users.

## Background

Tumorigenesis is a multidimensional issue involving genomic changes. It is also activated by cell-extracellular matrix (ECM) interactions between scaffolds and cytoskeletal structures [1–4] expressed via stressing of mechanosensors, similar to integrins, from multipart cellular forces capable of altering genomic programming [5]. The interactions of tumour microenvironment with ECM scaffolds usually activate cell’s membrane focal adhesion proteins and transmembrane signal receptors (TSR), epidermal growth factor receptors (EGFR), vascular endothelial growth factor (VEGFR) or nerve growth factor receptors (NGFR). The mechanosensors regulate tumour cell growth via signal transaction between the extracellular active domain of cells [6–9] and the intracellular F-actin filaments, by triggering an avalanche of phosphorylation reactions.

Protein conformational changes and excitation of TSR pathways requires the activating force to lay in the pN force range, and certainly below the nN gauge [10]. Besides random mechanical stressing and active chemical affinity strength, the binding efficiency (strength of bonding) between nanoparticles (NPs) and the proteins of the cell membrane can be modulated either via short or long-range electrical polar or other types of dispersive interactions. On the limited surface area of NPs, only a certain number of proteins can be attached for long enough to be biologically active [11], and space-confined local interactions with the biological milieu was recognised to be responsible for a set of diverging cell functionality routes [12]. Consequently, the signal transaction pathways from protein-NPs interaction are signalling out safety issues for NPs [11, 13].

As either favourable or adverse response in cells from NPs is type specific [11], the link between NPs and biological marks should be established on a case-by-case basis [14, 15].

Contradicting results from tumour cells exposed to NPs, for either ablative or tumour growth efficiencies or variable toxicity levels of NPs [16, 17], are also surfacing the safety issues. Nevertheless, despite progress, today there is a lack of knowledge on the specific pathways by which NPs interact with eukaryotic cells, precluding the identification of a universal NP therapeutic approach. Because different size NPs and diverse surface chemistries usually divert cellular responses, including NPs–membrane receptor binding and TSRs activity, the toxicity of NPs is related with the morphology of surfactants, the electrical charging state, the concentration and composition of proteins and nanomaterials in ECM [18–21] and finally the strength of molecular bonding between NPs and the cell phenotypes [22].

Previous studies of melanoma and cervical carcinomas exposed to silica, gold NPs and carbon nanotubes recognised that NPs size activates tumour cell growth selectively [23–27]. The correlation between TSR signals in human SK-BR-3 cancer cell line and the size of modified gold and silver NPs demonstrated that although 2–100 nm size NPs reformed signal transduction, an immense difference in apoptotic activity was attained when cells interacted with 40–50 nm sized NPs [26]. Recently, it was also suggested that changing the size of gold NPs from 5 to 40 nm, the growth rates of A549 and 95D cancer cell lines were conceivably tuned. Specifically, 5 nm sized NPs inhibited any proliferation of both cell types, while ~ 10 nm sized NPs did not have any effect on cell growth [27]. Likewise, A549 and THP-1 cells exposed to SiO_2_ NPs displayed size-dependent cytotoxicity as well as 15 nm sized NPs were also correlated with high cytotoxicity levels. On the contrary, 60 nm sized NPs exhibited lower toxicity. Finally, 200 nm sized NPs increased stem cell growth through ERK1/2 activation, whereas 2–4 μm sized NPs were able to activate different signal transduction pathways [28]. Small-sized NPs conjugate the EGFR and switch on the protein kinase B (AKT) and extracellular signal-regulated kinase (ERK) signal transaction pathways that inflame cell’s growth.

Rare-earth nanoparticles (RE-NPs) may also interact with specific domains such as metal ion-dependent adhesion sites (MIDAS), adjustment to MIDAS (ADMIDAS), synergistic metal ion binding sites (SyMBS) and ligand adhesion binding site (LABS), located in the α_ν_β_3_ subunit or other integrins subunits [29, 30].

Likewise, RE-NPs entail an additional degree of suppleness in tumour NPs interaction [31–34]. While ceria NPs (nanoceria) displayed a protective action against cellular damages by different radicals [35], low concentration levels of modified ceria NPs highly boosted of hepatoma cell proliferation by reducing apoptosis via activation of AKT/ERK signalling pathways [36]. Typically, an ensemble of NPs surrounding the cell is liable for cytoskeletal stressing and equally stand up for chemical, nano thermodynamic (Hill) [37], entropic or an electrical dipole interaction between NPs and mechanosensors. However, until now a casing of understanding of interactions between NPs, TSRs and cells remains vague and unavailable.

In principle, the strong ionic character of RE compounds should stimulate the mechanosensors of cells via electrical interactions. Also, because RE ions are widely used in different applications, it is vital to look into their potential contribution to tumour cell growth for implying appropriate public health protection protocols. Lanthanum fluoride (LaF_3_) and praseodymium fluoride (PrF_3_) are used in fluorescent lamps, radiation colour glasses, fibre optics, enamel’s applications and electrodes. LaF_3_ is elaborated in a specific type of glasses, phosphor lamp coatings, water treatment and catalysts. It is also an essential component of a commercial fluoride glass (ZBLAN), which mixed with europium fluoride is used for optical communications and as crystal membrane in ion-selective fluoride electrodes with a good transmittance in the infrared. Equally, PrF_3_ is also employed in carbon arc lights for the motion picture industry, studio lighting and projector lights. Fluoride glasses doped with praseodymium are also used in single-mode fibre optical amplifiers.

Thereby, this work demonstrates that tiny size RE-NPs had the potency to stimulate tumour cell growth via electrical dipole interactions.

The article is organised into three sections. First, the size distribution, the interactions and the geometry of NPs are analysed by applying dynamic light scattering (DLS), atomic force microscopy (AFM), transmission electron microscopy (TEM), X-ray diffraction (XRD), two-dimensional fast Fourier transform (2D-FFT) analysis and vacuum ultraviolet spectroscopy (VUV 110–180 nm). Next, the correlation between the level of growth of three different human cancer cell lines (A549, SW837 and MCF7) with the size distribution and the concentration of LaF_3_ and PrF_3_ NPs is established. Finally, within a requisite force limit of 1 pN for activating mechanosensors and subsequently tumour cell growth, the viability of tumour cells is fitted within a theoretical motif of electrical dipole interaction between one RE-NP and one LABS. The work contributes towards identification and classification of different types of cytoskeletal stressing and interactions between NPs and mechanosensory of cancer cells.

## Results

### Size and Structure of NPs

**Fig. 1:**
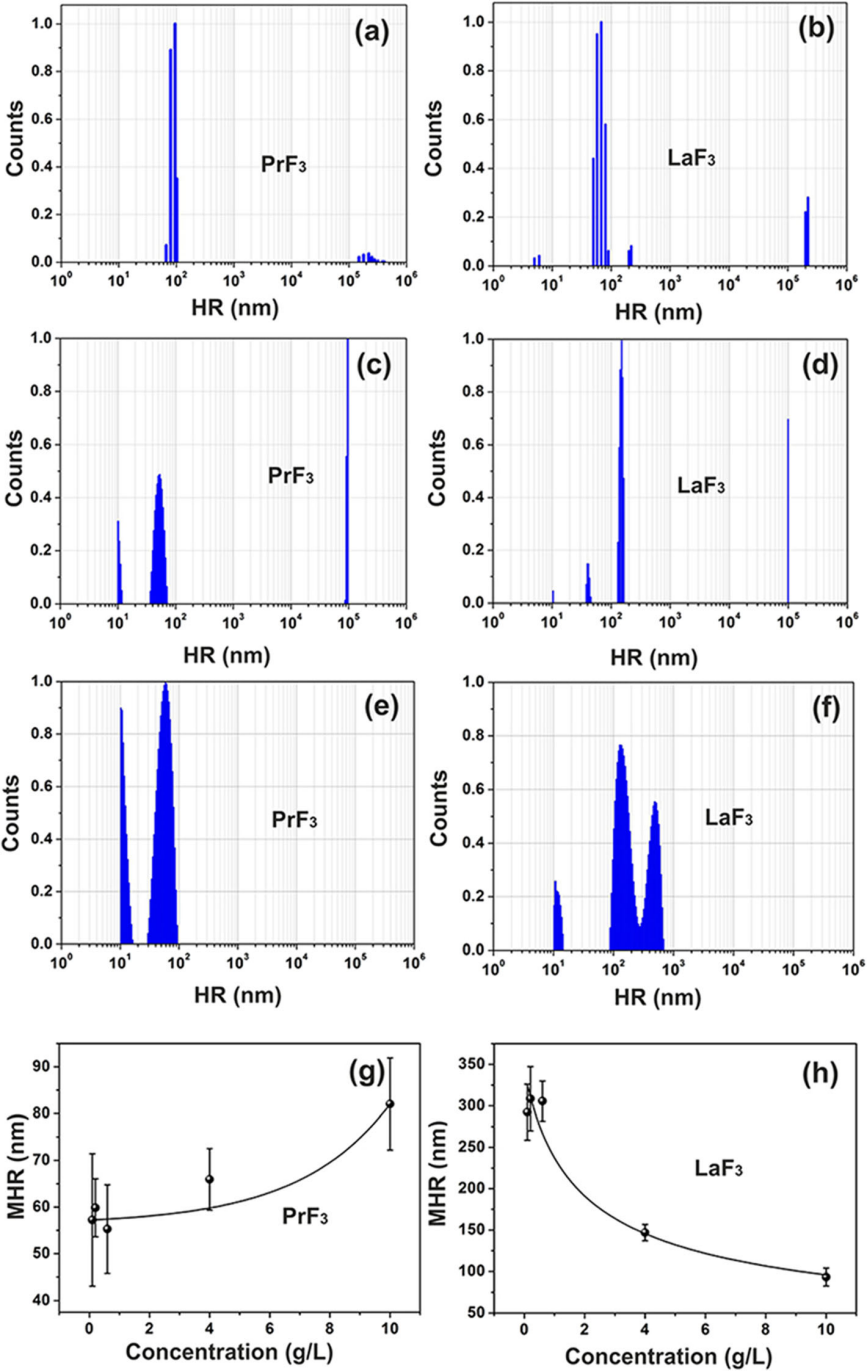
DLS size distribution spectra of RE suspensions. **a, b** PrF_3_ and LaF_3_ NPs (5 g/L) in water. **c, d** PrF_3_ and LaF_3_ NPs (5 g/L) in DMEM+FBS. **e, f** PrF_3_ and LaF_3_ NPs (0.1 g/L) in DMEM+FBS. **g, h** Mean hydrodynamic radius (MHR) with standard deviation of PrF_3_ and LaF_3_ NPs in DMEM+FBS at different concentration levels

**Fig. 2:**
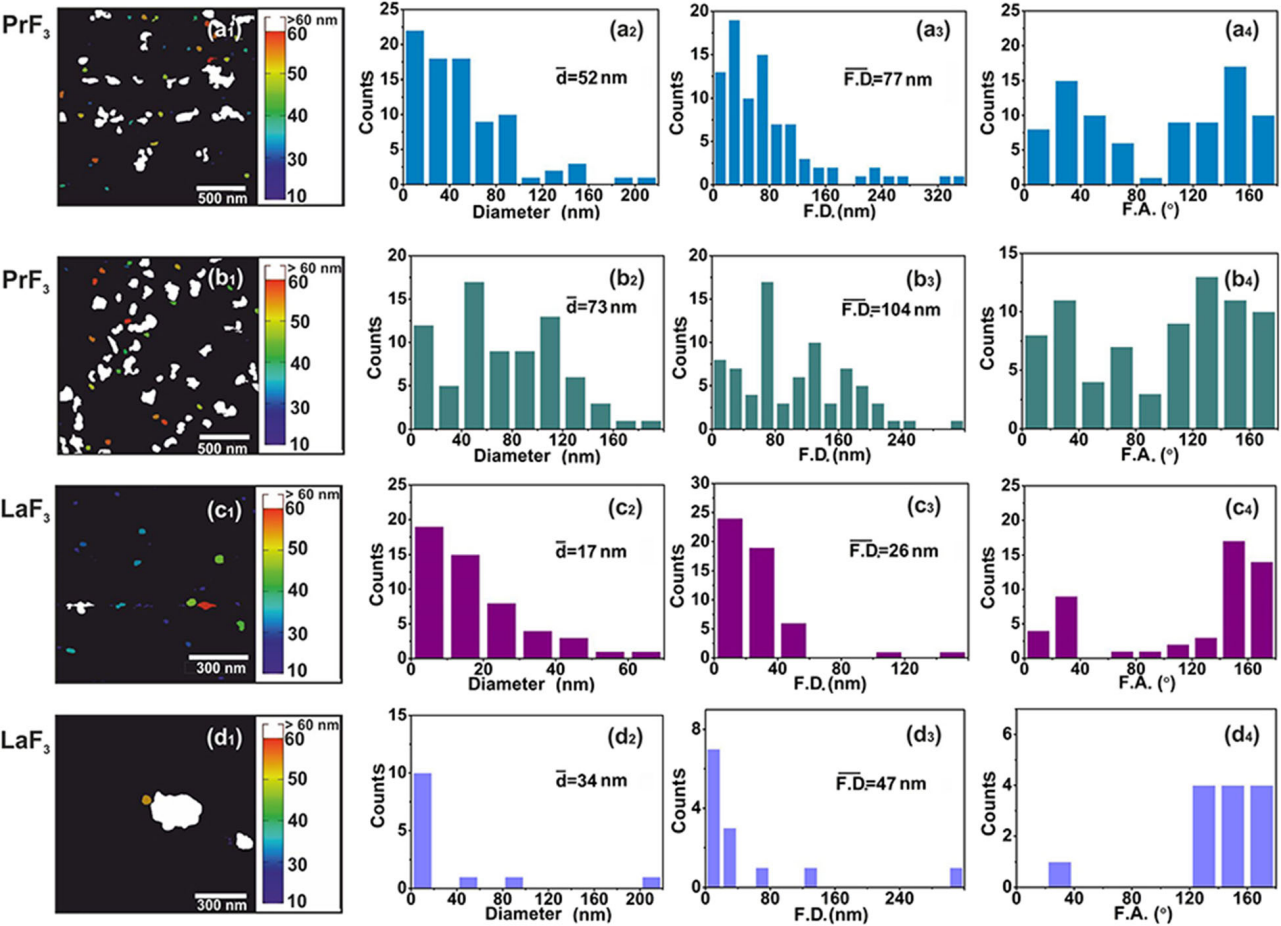
AFM digital (*x*, *y*) size histograms of RE-NPs. (**a1–d1**) PrF_3_ (2 × 2 μm^2^) and LaF_3_ (1 × 1 μm^2^) NPs in DMEM+FBS suspensions. (**a2–d2**) (*x*, *y*) size histograms of mean equal circle area diameter $\left(\overline{d}\right)$ of RE-NPs. (a3–d3) (*x*, *y*) size histograms of Feret area diameters $\left(\overline{\ F.D.}\right)$ of RE-NPs. (**a4–d4**) Feret angle (F.A.) histograms relative to the *x*-axis. RE-NPs were oriented along two main directions between ±(44–60°)

**Fig. 3:**
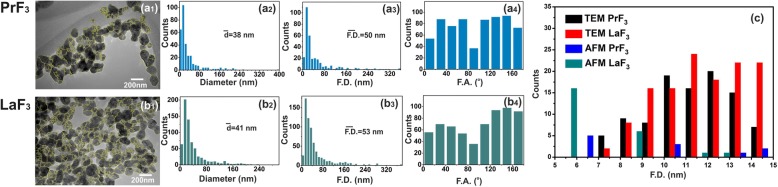
TEM digital (*x*, *y*) size histograms of RE-NPs. (**a1, b1**) TEM images of RE-NPs. Yellow lines indicated 2D boundaries between RE-NPs. (**a2, b2**) (*x*, *y*) size histograms of mean equal circle area diameter $\left(\overline{d}\right)$ of RE-NPs. (**a3, b3**) (*x*, *y*) size histograms of Feret area diameter $\left(\overline{\ F.D.}\right)$ of RE-NPs. (**a4, b4**) Feret angle (F.A.) histograms relative to the *x*-axis with preferential directions at ±(44–60°). (**c**) Size histogram of Feret diameter of small and tiny size RE-NPs extracted from both AFM and TEM images for 4 μm^2^ areas

**Fig. 4:**
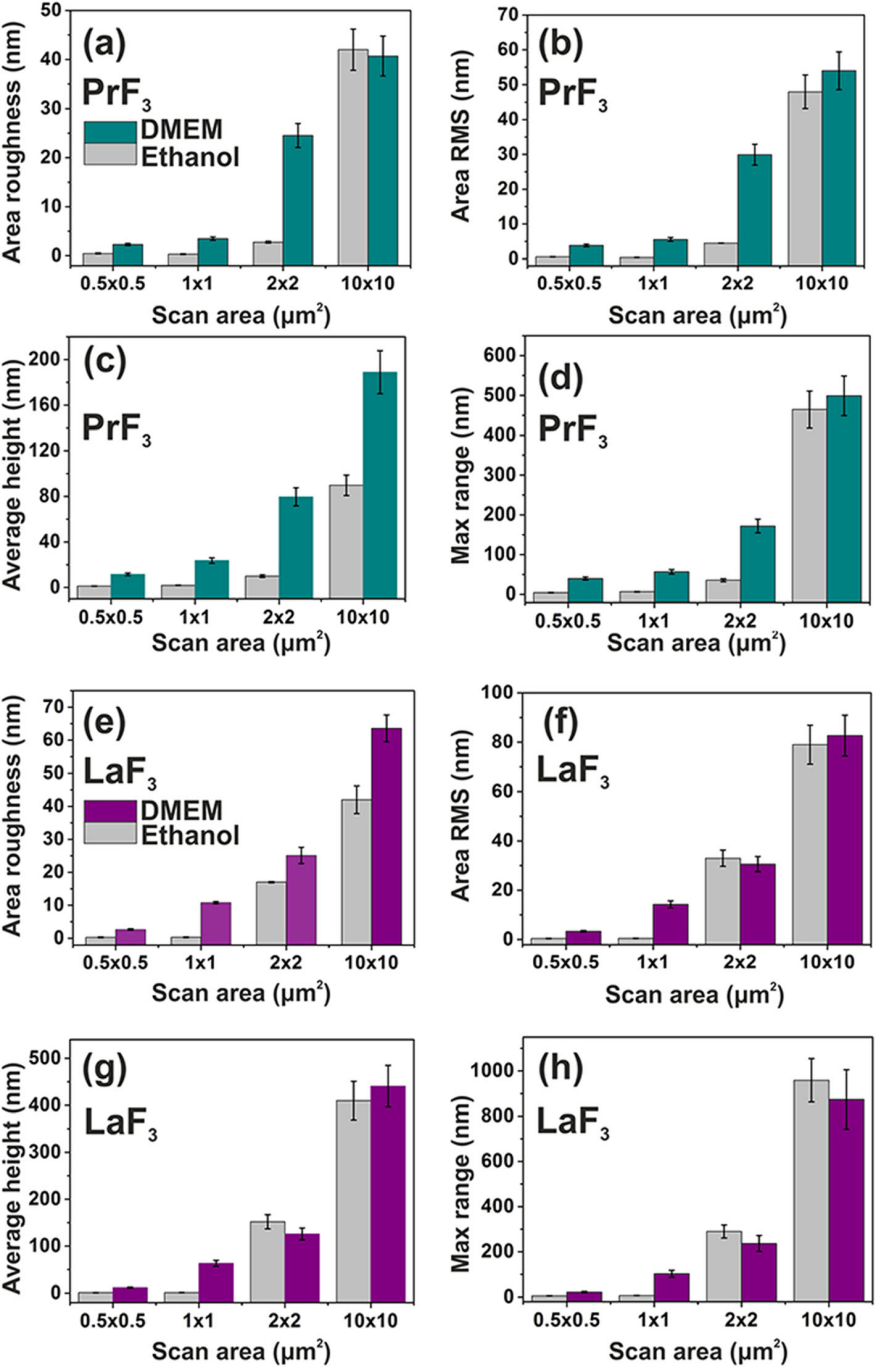
Surface parameter histograms (z-height) of PrF_3_ and LaF_3_ RE-NPs on a glass substrate in DMEM+FBS and ethanol for different scanning areas. **a**, **e** Area roughness. **b**, **f** Area RMS. **c**, **g** Average height. **d**, **h** *Maximum z*-range

**Fig. 5:**
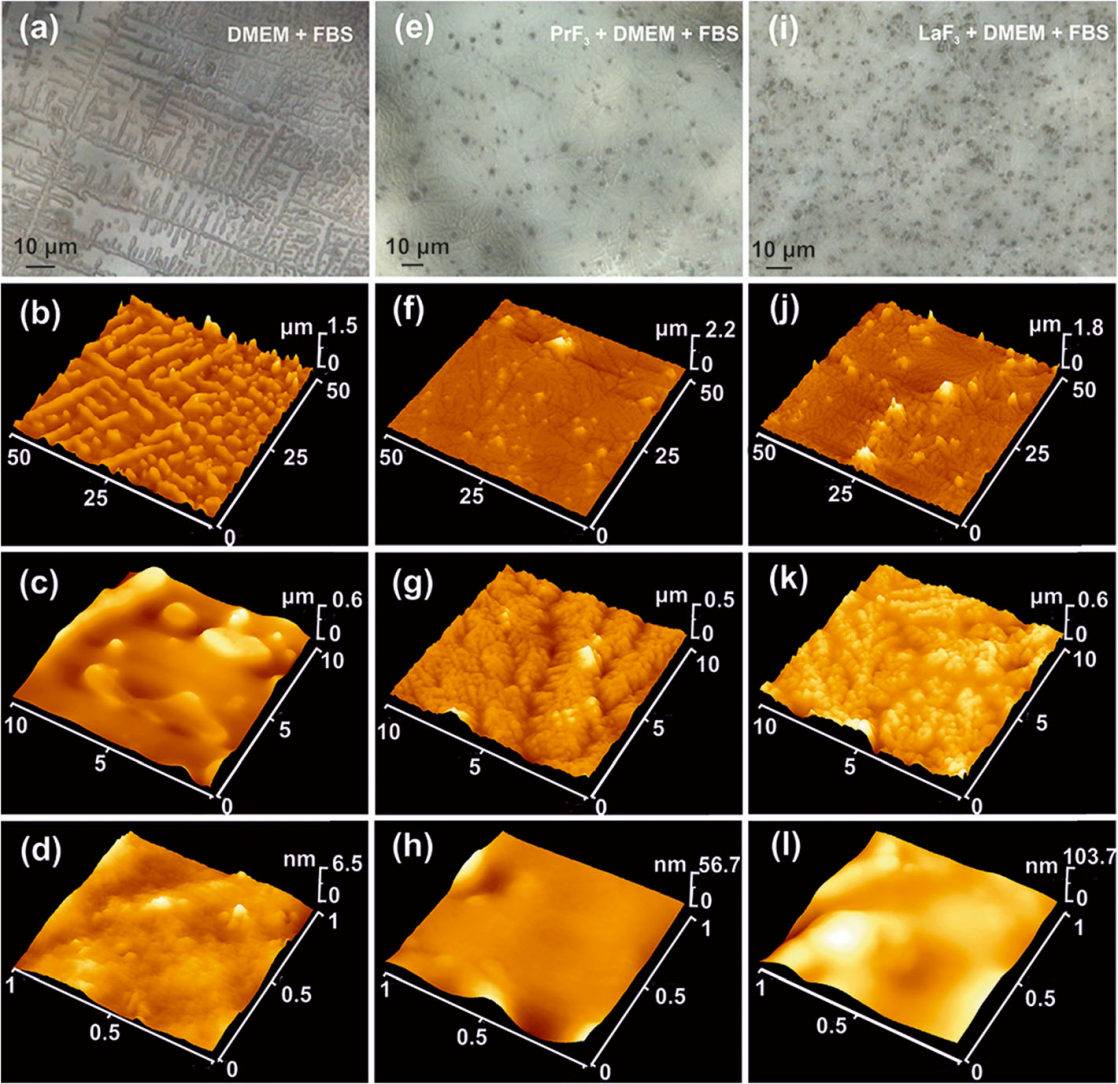
AFM and optical images of dried RE-NPs in DMEM+FBS. **a**–**d** Optical (**a**) and AFM images (**b**–**d**) of DMEM+FBS showing 5 μm self-assembled structures. **e**–**h** CCD (**e**) and AFM images (**f**–**h**) of PrF_3_ NPs in DMEM+FBS media showing 500 nm dendrite-self-assembled structures. **i**–**l** CCD (**i**) and AFM images at different magnifications (**j**–**l**) of LaF_3_ NPs in DMEM+FBS showing 100 nm dendrite-self-assembled structures

**Fig. 6:**
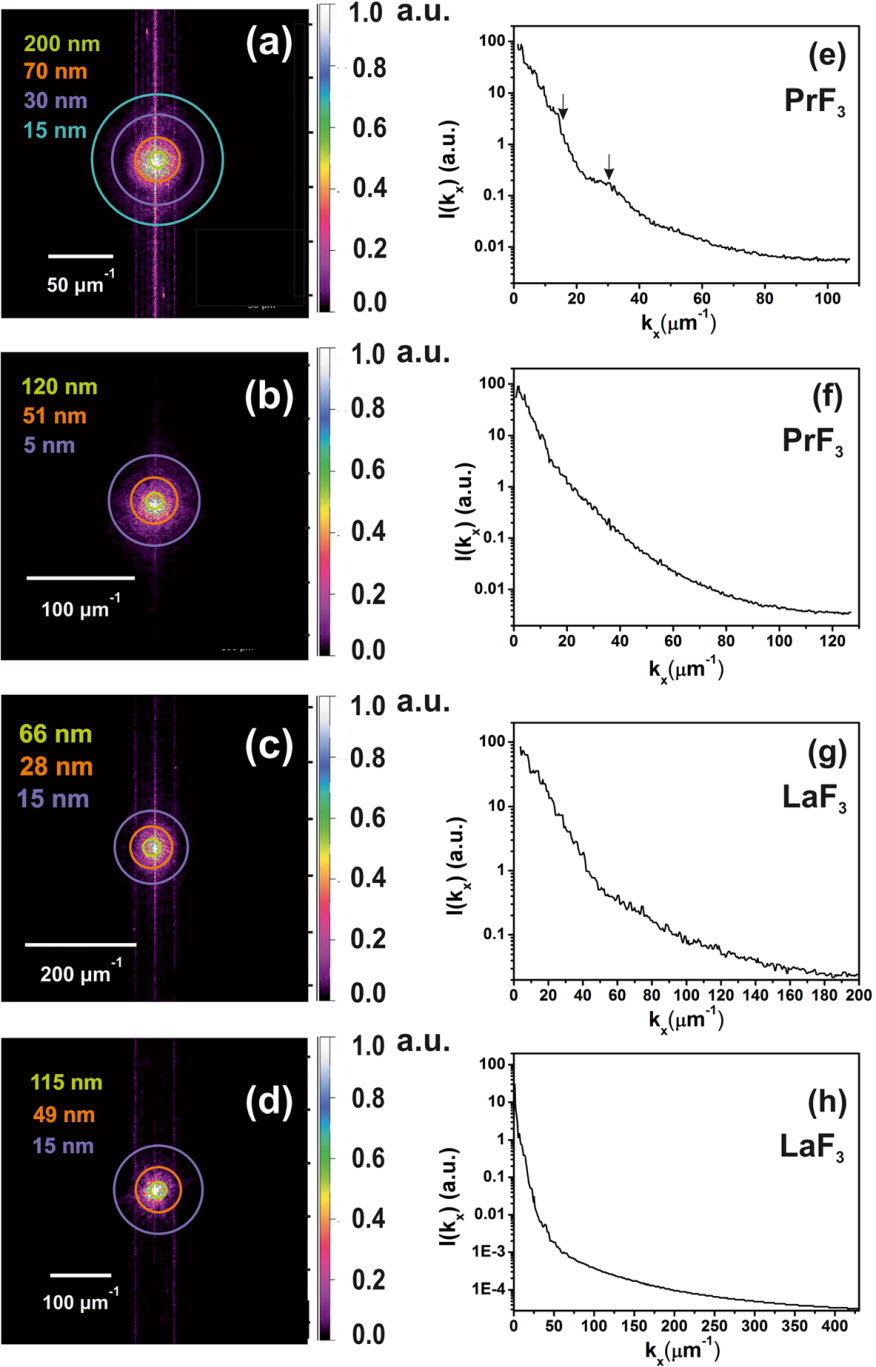
2D-FFT spectra of *z*-height distribution of dried RE-NPs in DMEM+FBS media evincing the presence of small-sized NPs in the liquid suspensions. **a**, **b** z-height distribution of PrF_3_ NPs from AFM images, Fig. 2(a1, b1). Small *z*-height features (~ 5 nm) were identified in (**b**). **c**, **d** z-height distribution of LaF_3_ NPs from AFM images, Fig. 2(c1, d1). **e**, **f** Power spectra of *z*-height wave vectors of PrF_3_ NPs along the *x*-axis. **g**, **h** Power spectra of *z*-height wavevectors of LaF_3_ NPs along the *x*-axis

**Fig. 7:**
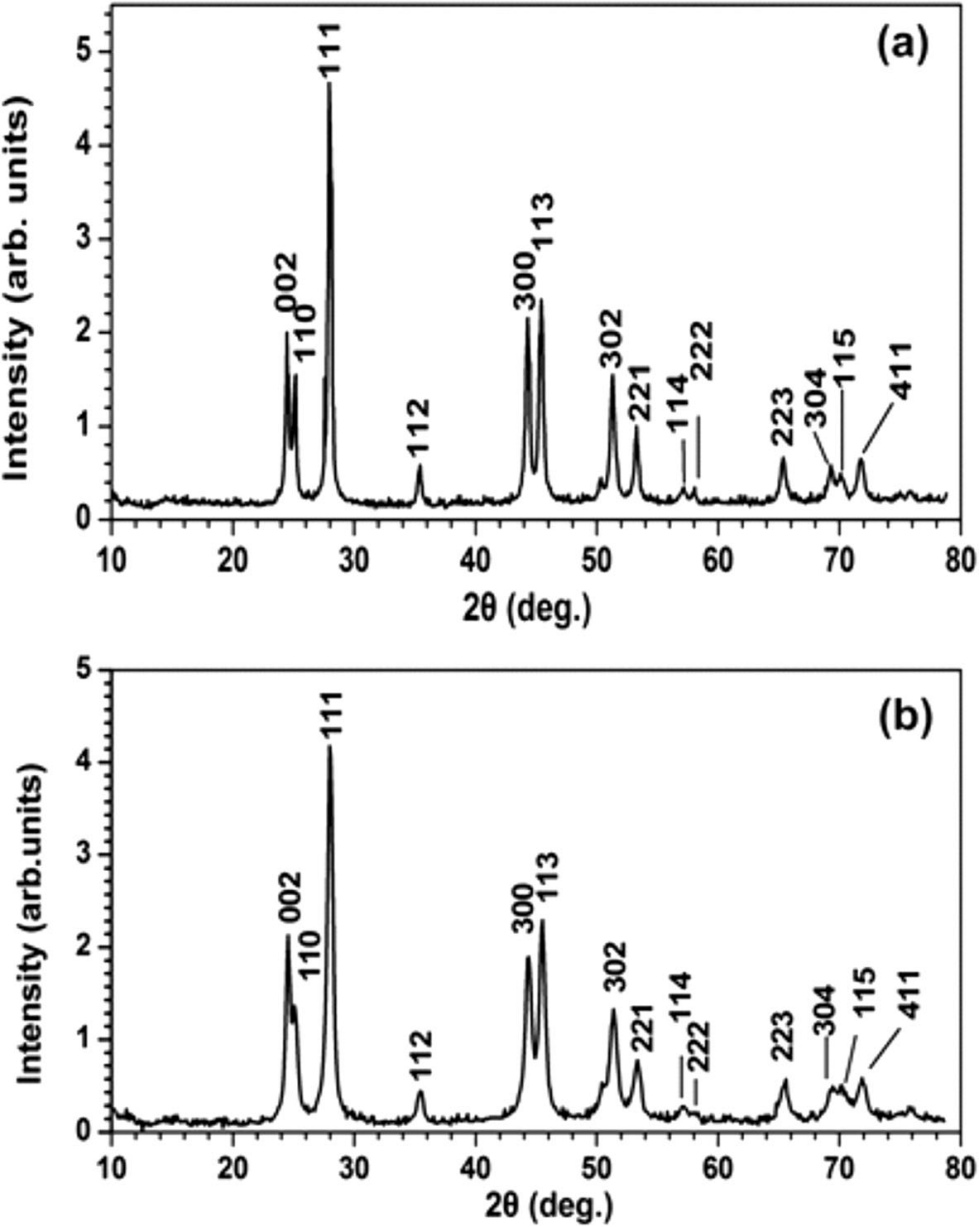
XRD spectra of **a** PrF_3_ and **b** LaF_3_ NPs. The mean surface area diameters of PrF_3_ and LaF_3_ NPs were ~ 23 ± 4 and ~ 15 ± 4 nm, respectively

First, DLS, AFM, TEM, XRD, FFT, VUV spectroscopy and *t* test statistics were applied to extract the size distribution of RE-NPs in liquid suspensions (Figs. 1, 2, 3, 4, 5, 6 and 7). Next, viability tests of cells and Western blotting (Wb) assays were used to identify the activation of the specific mechanosensors by RE-NPs.

### DLS

Cloudy heterogeneous mixtures were initially formed by adding RE-NPs in cell-culture media, evincing the complexity of interactions between RE-NPs in liquid suspensions. Tiny (< 10 nm), small (> 10 and < 20 nm) and large-sized (> 20 nm) structures of RE-NPs were identified for both PrF_3_ and LaF_3_ solubles (Fig. 1a–d).

The mean hydrodynamic radius (MHR) values of large size RE-NPs (55–83 nm for PrF_3_ and 99–296 nm for LaF_3_) were trailed directly and inversely proportional to the concentration level of NPs (0.1–10 kg m^−3^) in liquid Dulbecco’s modified Eagle’s with fetal bovine serum (DMEM+FBS), Fig. 1g, h. Also, the MHR of small size LaF_3_ and PrF_3_ NPs remained constant, 10.66 ± 0.74 nm and 10.64 ± 0.40 nm, respectively, at different concentration levels of RE-NPs. The MHR of RE-NPs remained unchanged for at least 6 days. After drying the RE-NPs suspensions, it was impossible to re-dissolve the RE powder again, as large size agglomerations stabilised by strong interactions forced precipitation.

### AFM and TEM Imaging of RE-NPs and Surface Analysis

For a reliable size distribution and statistics of small-sized RE-NPs in DMEM+FBS at 0.1 kg m^−3^ AFM (scan areas 1 × 1 and 2 × 2 μm^2^) and TEM imaging were also applied (Fig. 2(a1–d1) and Fig. 3(a1, b1)). After transferring the liquid drops of the RE-NPs in DMEM+FBS to the glass substrate, a relatively large number of non-aggregated tiny RE-NPs was identified [38] from both the mean size and the mean Feret diameter of the NPs (Fig. 2(a2–d2, a3–d3) and Fig. 3(a2, b2, a3, b3)). Also, the histograms of the angle distribution of AFM and TEM Feret diameters (for the larger dimension of NPs) indicated that both RE-NPs were oriented preferentially along two directions between ± (44–62^o^), relative to the *x*-axis (Fig. 2(a4–d4) and Fig. 3(a4, b4)).

Despite that *z*-height distribution of NPs did not provide any direct information on the overall NPs size distribution, it is a handy comparative tool for the first estimation of (*x*, *y*) size distribution because the z-height and (*x*, *y*) distributions stay interrelated [38].

The mean surface parameters of both PrF_3_ and LaF_3_ in dried suspensions for different AFM scan areas are also shown in Fig. 4. The small *z*-height values indicated a very uniform *z*-height distribution of both RE-NPs for small 1 × 1 μm^2^ scan areas. On the contrary for both RE-NPs and larger scan areas, the *z*-height distribution was significantly broader. The low *z*-height distribution values at small scan areas reflect the presence of small-sized RE-NPs in the liquid suspensions. The surface parameters values in DMEM+FBS medium were on the average larger than in ethanol, showing a complex reactive state between proteins and RE-NPs, in agreement with the multifaceted structuring of Fig. 5 and the 2D-FFT data (Fig. 6). Overall, LaF_3_ NPs exhibited an intriguing response in dried suspensions and more extensive surface roughness parameters than PrF_3_ NPs.

### FFT

Coloured rings were added in selected radii in the 2D-FFT spectra (Fig. 6a–d). The cycles represent different NPs size distribution in the Euclidean space, from a tiny size equal to a pixel’s size (1.9–3.9 nm) to a considerable size of ~ 2 μm, which was the upper scanning limit of the AFM tip in the *z*-axis (Fig. 6e–h). A deconvoluted of the *z*-height values with the radius of the AFM tip provides an actual resolution in the *z*-height distribution of ~ 5 nm. The 2D-FFT spectra demonstrated an intense distribution of wave vectors near the centre, owing to a mean *z*-height of RE-NPs of ~ 44 nm. The FFT patterns exhibited a halo structure, which is smeared out gradually, because of a polydispersed broad tiny size structure identified in the 2D-FFT spectra. As only the halo appeared in the spectra without any diffractive patterns for both 2D-FFT spectra, regular self-assembled structures were missing. The characteristic correlation lengths obtained from the ring-like 2D-FFT patterns of PrF_3_ and LaF_3_ were ~ 51, 70 nm and 28, 49 nm, respectively, in agreement with the MHR values extracted from the DLS spectra.

### XRD

XRD spectroscopy characterised the crystal structure and provided complementary information on the size of PrF_3_ and LaF_3_ NPs (Fig. 7). The sharp diffraction peaks, corresponding to the standard hexagonal phase structure for both RE-NPs, reveal a high crystalline state of the agglomerating phases. By using the Scherrer formula ($\tau =\frac{0.9\lambda }{\beta \cos \left(\theta \right)}\Big)$, the average mean equal area circle diameter (MEAC) *τ* of PrF_3_ and LaF_3_ NPs was estimated to be ~ 23 ± 4 and ~ 15 ± 4 nm, respectively.

### VUV Spectroscopy

**Fig. 8:**
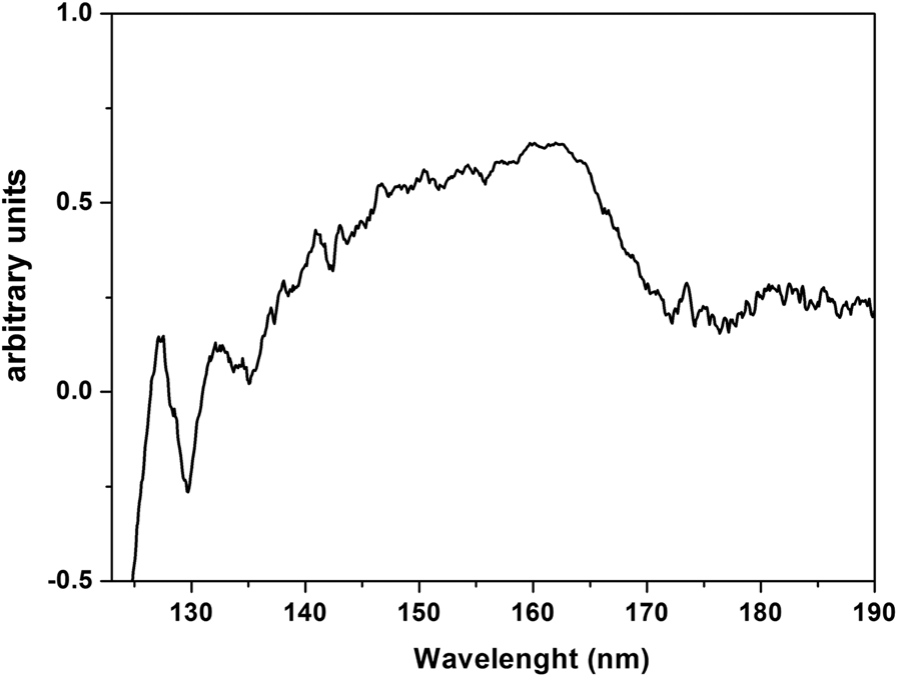
VUV transmission spectrum of PrF_3_ NPs in water suspensions deposited on the dried CaF_2_ substrate. The spectrum indicates water attachment and trapping within PrF_3_ NPs

The VUV transmission spectrum of the hydroscopic PrF_3_ NPs layer deposited on CaF_2_ substrate, from 125 nm (~ 10 eV) to 190 nm (~ 6.5 eV) is shown in Fig. 8. The VUV peaks at 140–170 nm previously attributed to the transitions of Pr^3+^ trivalent ions from the ground *4f* electronic state configuration to the Stark components of the *4f5d* electronic configuration inside YF_3_, LaF_3_, KY_3_F_10_ and LiLuF_4_ single crystal matrix and they are overlapped with a water VUV absorption band, revealing the presence of bound water molecules in the PrF_3_ and LaF_3_ crystals.

### Viability Test

Following size distribution analysis and statistics of RE-NPs, the water-soluble tetrazolium salts (WST) viability test was used to monitor the toxicity of PrF_3_ and LaF_3_ NPs for three human cancer cell lines, the A549 derived from lung cancer, SW837 derived from colon cancer and MCF7 derived from breast cancer. Three different concentrations of RE-NPs suspensions (0.5, 1 and 5 mM) in DMEM+FBS (A549, SW837) and Roswell Park Memorial Institute medium with Fetal Bovin Serum (RPMI+FBS) (MCF7) were used. The cell lines were initially placed on 96-well plates, left to attach overnight. To be within the linear region of cell growth and to avoid saturation (Fig. 9a) a day after, fresh medium containing PrF_3_ and LaF_3_ suspensions were added, and viability tests were performed 24 and 48 h later after the addition of RE-NPs, or 48 and 72 h after the initial moment of cell plating. However, for the three concentrations and the three culture cell lines, an overgrowth difference was detected, provided that the medium was not replaced and additional RE-NPs were not added in the culture, practises which alter the initial conditions of the experiment. Also, it was impossible to plate a cell concentration less than ~ 5 × 10^4^ cells per well because the confluence for the three cell lines was too small to guarantee a measurable cell growth. The optimum experimental set up was set up for ~ 5 × 10^4^ cells per well.

**Fig. 9:**
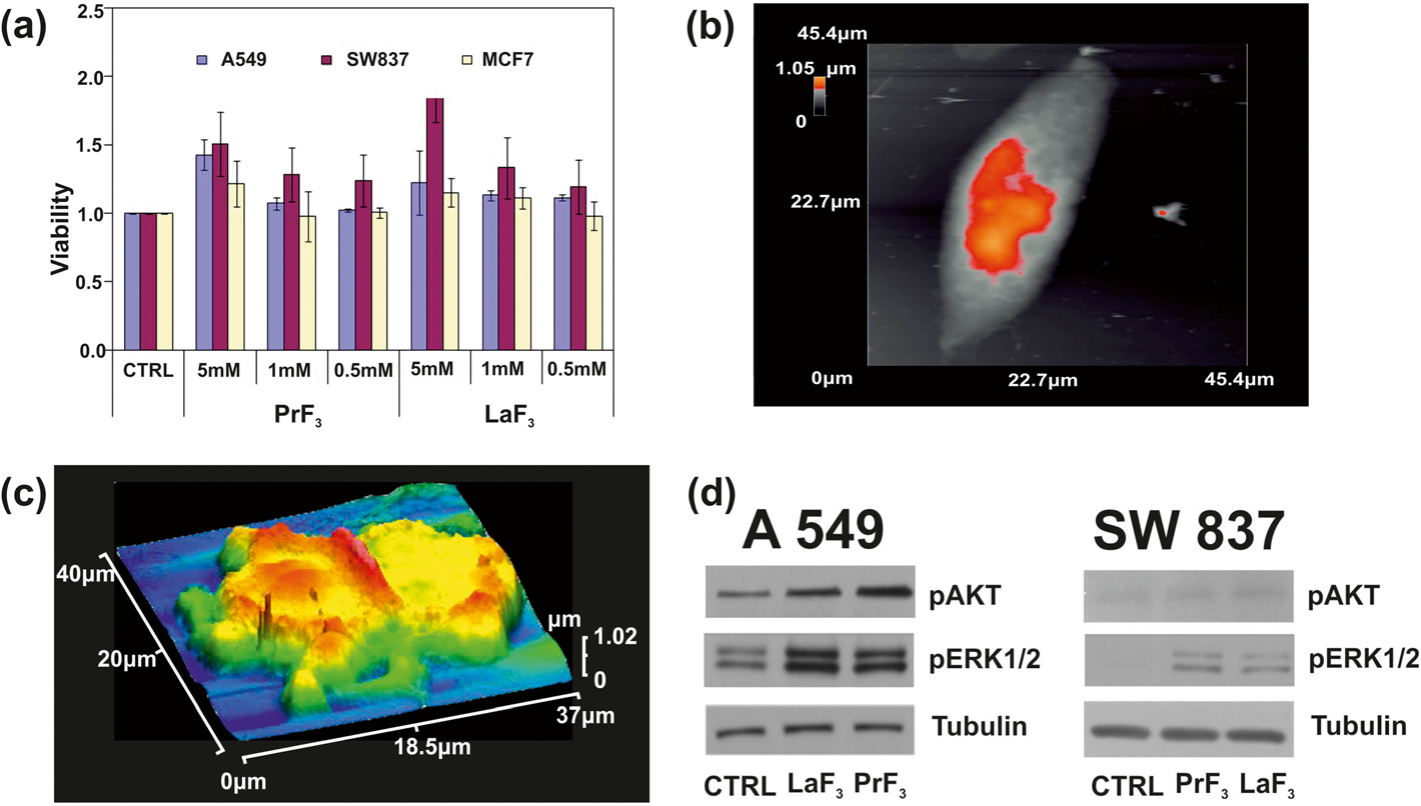
**a** WST viability assay histograms of three different cancer cell lines (A549, SW837, MCF7) treated with different concentrations of PrF_3_ and LaF_3_ NPs in biological media. **b** AFM image of a single A549 cancer cell. **c** AFM image of a divided A549 cancer cell in RE-NPs in DMEM+FMS. **d** Wb phosphorylation analysis of the A549, SW837 cells with AKT and ERK pathways

At higher concentration (5 mM), for both RE suspensions, an ascending growth for all cell lines was obtained (Fig. 9b, c). Among them, the highest growth value was for the SW837 line (86%, LaF_3_). Less pronounced, but still relevant, a cell overgrowth (15%) was noticed for the MCF7 cell line at 5 mM. A *t* test statistical analysis (*p* and Fisher *F* values) of tumour cells viabilities showed that the growth of tumour cells was un-saturated at 24 h; it followed an unknown physical law connecting viability and concentration of RE-NPs (Additional file 1).

### Phosphorylation Assays

The phosphorylation status of two proteins was also tested (Fig. 9d). Using specific antibodies and Wb assays in the A549 and SW837 cell lines grew in DMEM+FBS with 5 mM of LaF_3_ and PrF_3_ NPs for 24 h, high phosphorylation activity of ERK1/2 and AKT in treated cells, as compared to control cells (CTRL), was obtained.

## Discussion

The relative growth rate of cancer cells was ascending at higher concentration levels of both RE-NPs (Fig. 9a). However, the MHR values of RE-NPs in DMEM+FBS were trailed directly (PrF_3_) and inversely proportional (LaF_3_) to the concentration of RE-NPs of 0.1–10 kg m^−3^ (Fig. 1g, h). Therefore, RE-NPs with average size above ~ 55 nm should not have any effect on cell growth and only tiny size NPs can take an actual part in tumour growth.

### Size and Structure of RE-NPs

#### Identification of Tiny RE-NPs

From the experimental data, mean size, distribution and the statistical parameters of RE-NPs were extracted. By applying *t* test statistics for the “null hypothesis” of “mean equal area circle” of NPs in dried PrF_3_ and DMEM+FBS suspensions, the *p* value of the diameter of NPs between two randomly selected AFM images was ~ 0.001 (Additional file 2). A value of MEAC diameter (63 nm) was extracted with confidence from the AFM data, and this was comparable with the MHR value from the DLS data (Fig. 1g).

On the contrary, the value of the MEAC diameter from randomly selected LaF_3_ samples showed an average MEAC diameter of 26 nm with a higher rejection probability value (*p* = 0.07), pointing to a diverging behaviour of LaF_3_ in liquid suspensions. The discrepancy between the MEAC diameter and the MHR from DLS (296 nm) (Fig. 1) is owing to the complexity of interactions in LaF_3_ suspensions again. Indeed, for a 2 × 2 μm^2^ AFM tip scanning area, the average *z*-height was ~ 140 nm, displaying the presence of large size LaF_3_ NPs, transferred from the liquid suspensions on the substrate (Fig. 4). For the “null hypothesis” of “equal MEAC diameter values from randomly selected TEM samples”, the *p* values were also small (*p* = 0.001). For both RE-NPs the average MEAC diameter values extracted from the combined TEM and XRD data for both PrF_3_ and LaF_3_ indicated high *p* values, *p* = 0.29 and 0.06, respectively, not allowing thus any correlation between the TEM and XRD data. Only TEM, AFM (PrF_3_) and DLS data were sufficiently reliable to extract MEAC diameter and core-shell values (Additional file 2).

Also, a non-isotropic angle distribution of Feret diameters indicated that both PrF_3_ and LaF_3_ structures were highly polarised dielectrics, as the anisotropic angle distribution is an indication of strong electrical polar interactions between nanocrystals. A diverse polarised state of LaF_3_ was responsible for scaling down the relative efficiency of the agglomerating state in suspensions and an upscaling of the surface roughness parameters in dried samples.

A software particle analysis of random AFM images for 5 μL and a concentration of 0.1 kg m^−3^ identified a number of ~ 22 and ~ 11 RE-NPs with size smaller than 15 nm and 10 nm (*p* = 0.001), respectively, and a number of ~ 60 RE-NPs from TEM images (*p* = 0.001) in an area of ~ 4 μm^2^, confirming thus the presence of tiny size RE-NPs in the suspensions (Fig. 3(c), Additional file 2) not detected with DLS.

#### Structure and Geometry of RE-NPs

The size distribution of RE-NPs is divergent in ethanol and DMEM+FBS suspensions (Fig. 4). The diversity is owning to different molecular interactions between the adsorbed proteins, carbohydrates, electrolytes and the surface of RE-NPs, leading to the formation of highly complex organic cloaks (corona), which modulate the specific interactions of RE-NPs’ with cells in DMEM+FBS medium.

The interplay between water molecules trapped in hygroscopic RE-NPs and DMEM+FBS was also vital for core-shell formation. It also had a profound effect on protein and conformational changes in the in-between interactions during the initial phase of preparation. As the surface-to-bulk ratio of NPs developed high values in the suspensions, the effective stability and the physicochemical, mechanical and flow properties of RE-NPs, including the ability to absorb proteins, were exceedingly varied [39–41].

Comparative size distribution of RE-NPs in liquid (DLS) and solidified suspensions via AFM and TEM showed that RE-NPs were encapsulated inside organic shapes forming core-shell dielectric structures, where a proteinaceous shell surrounds the RE-core. The AFM imaged from solidified RE-NPs in DMEM+FBS suspensions deposited on glass substrates also point to the formation of multifaceted RE-NPs and protein corona complexes (Fig. 5). While dried media formed a regular self-assembled patterning of crystal structures (Fig. 5a–d), dried RE-NPs suspensions showed an amorphous layered structure having several black spots, visible even with the AFM’s digital camera (Fig. 5e–l). At higher optical magnification, discrete agglomerations of globular shapes, smaller than those in the medium alone, were also detected for both RE-NPs in DMEM+FBS, together with dendrite-type structures, both showing the complexity of interactions, in agreement with the surface parameter results (Fig. 4). Even with the highest AFM resolution (1 × 1 μm^2^ area), the last lane of Fig. 5, no isolated RE-NPs aggregations, within the resolution limit of ~ 5 nm, were identified in dried structures for both RE-NPs. The black colour spherical mycelia, 1–2 μm long, shown in the optical images, were large agglomerating formations of core-shell RE-NPs. The complexity of reactions between the RE-NPs and DMEM+FBS was visualised via the transformation of the long-term self-assembled elongated structures in pure DMEM+FBS to dendrite structures.

The results point to the picture of single RE-NP core structure encapsulated inside a protein shell. These structures were undetectable because they were surrounded by organic matter and electrolytes, both cross-reacted with RE-NPs. The VUV spectrum of PrF_3_ shows some spectral peaks between 140 and 170 nm (Fig. 8). The ionic transitions are overlapped by a VUV water absorption band extended from 145 to 180 nm with a *maximum* at 168 nm. Only the spectral signatures of the *4f6s* electronic configuration with *maxima* at 132 and 127 nm were present in the spectrum. However, these bands could evince the presence of water in the high hygroscopic PrF_3_ suspensions. Water has a rich, structured absorption band in the VUV spectral range centred at 122 nm, revealing the presence of water molecules in the core-shell NPs.

### Activation of Mechanosensors

#### Activation of Integrins by External Forces

**Fig. 10:**
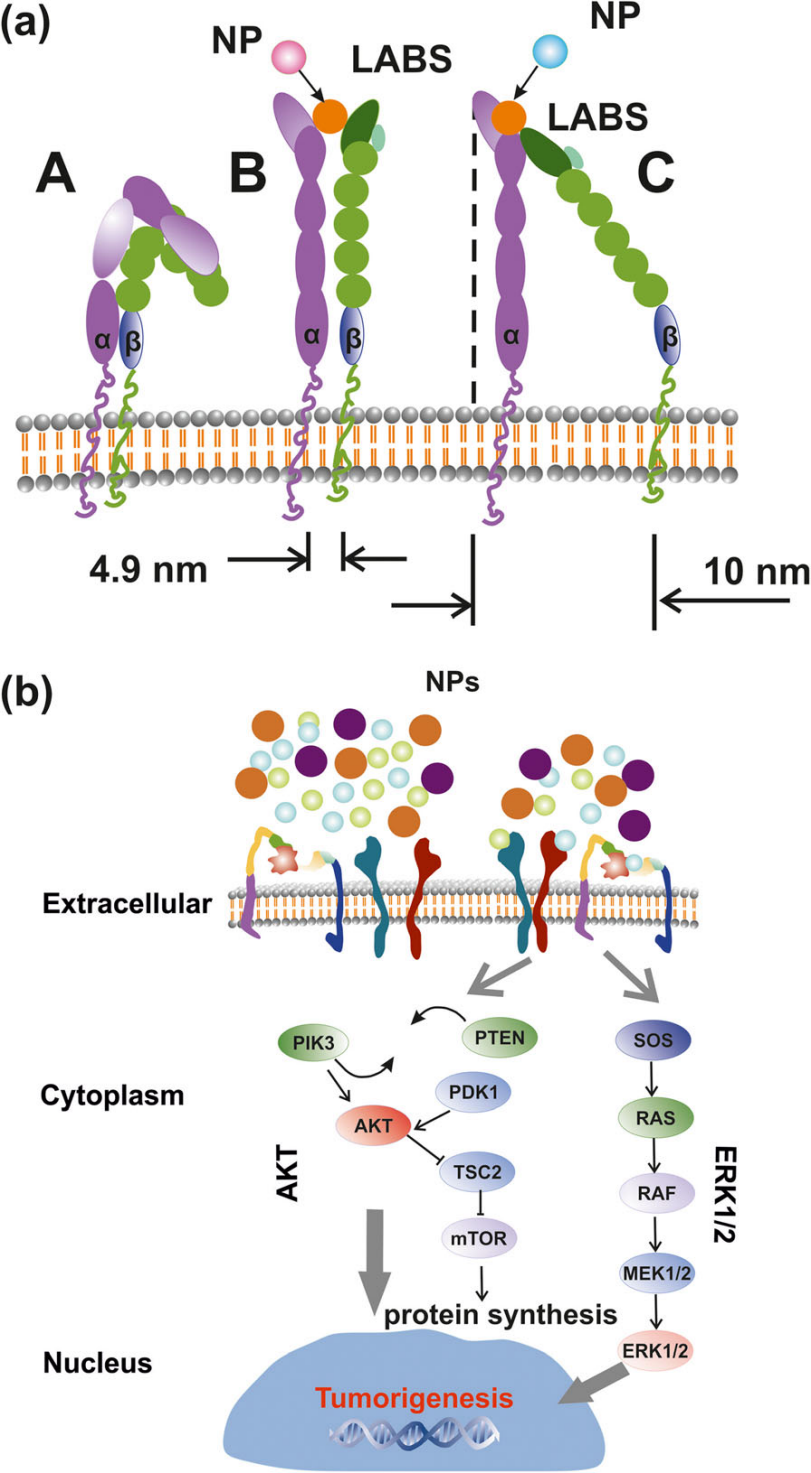
Simplified layout of integrin activation by NPs and signal transaction pathways. **a** Structure and conformational geometries of integrins at a low (A), medium (B) and high-affinity strengths (C). **b** AKT and ERK1/2 signal transaction pathways activated by RE-NPs via external integrin stimulation

The activation of oncogenic pathways by RE-NPs [24], besides the 3D structural nature of TSRs, is based on some Natural Evolution principles for sustaining the viability of cells. First, upon binding a specific external ligand in a LABS, conformational changes along the entire TSR spectrum underline a series of cascading pathways, triggering tumour cell growth (Fig. 9c, d). The transmission of signals advances through the plasma membrane via various protein chains. Signal transduction was via conformational transformations of integrins responding to a high affinity external force (Fig. 10a, b).

Because of “life sustainability” and “survival laws” that prevents cancer cell growth by random “noise”, it is required that the strength of the external force should be within a bounded range of values and also the external strength stimulus should apply for a long period on a large number of mechanosensors in a cancer cell. The external strength that stimulates cancer must be slightly larger than the strength of the interatomic molecular forces under normal conditions. For a thermal energy of a ligand at room temperature (*kT* = 0.025 eV, *T* = 298 K), and for a regular thermal stress of molecular bonds of ~ 0.05 nm, the mean thermal force acting on the LABS stays for 1.2 × 10^−12^ N. In principle, a force above ~ 10 × 10^−12^ N acting coherently on the whole set of mechanosensors on a cell should activate signal transduction in tumour cells. Consequently, ignoring any thermal and mechanical stressing in the ECM normal conditions, integrin activation via electrical polar interactions between LABS and NPs has the potency to start signal transduction in cancer cells and to initiate tumorigenesis.

#### Integrin Structure and Geometry

An integrin receptor in the upright conformation state extends ∼ 20 nm upwards from the cell membrane [42] (Fig. 10a). For no contacts between the two α- and β-subunits, other than those in the headpiece near the ligand-binding pocket, the α- and β-subunits are well separated with their cytoplasmic tails extended out up to ∼ 8 nm [42]. A conic projection geometry (20 nm slant height, 5–10 nm diameter of its circular base), bounded by the α- and β-subunits, defines a projected area on the surface of cell’s membrane between ~ 19 and ~ 80 nm^2^, for a typical mean radius of a tumour cell *R*_*c*_ ≈ 5 μm (equivalent surface area of a spherical cell ${S}_c=4\pi {R}_c^2=3.14\ \mathrm{x}\ {10}^8\ {\mathrm{nm}}^2$). By dividing the area *S*_*c*_ of a spherical tumour cell surface with the projected area of an integrin on a cell surface, an upper limit of the number of integrin receptors for these projected areas was *n*_*int*_ = 1.6 x 10^7^ and 3.9 × 10^6^ respectively. These numbers are compared with the mean number of integrins on a cell ${\overline{N}}_{int}\approx 2\ \mathrm{x}\ {10}^5$ and for an average interspacing of 45 nm between adjacent integrin receptors [43]. Nevertheless, ${\overline{N}}_{int}$ might be larger because of an uneven surface structure, different separating distances between integrins and variable size of tumour cells (Fig. 9c), but the number of integrins on a cell membrane stand between *n*_*int*_ and ${\overline{N}}_{int}$.

### Interaction of Mechanosensors with RE-NPs

#### ERK ½ and AKT Activation

**Fig. 11:**
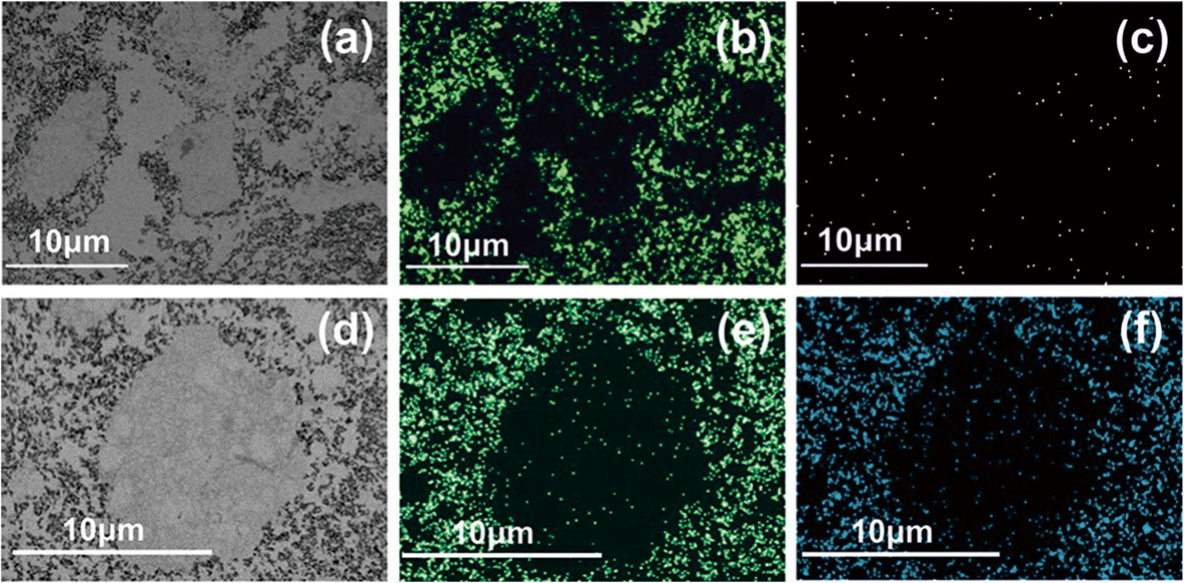
TEM images and elemental analysis of RE-NPs at the surface boundaries of A549 cells. **a** TEM image of small size LaF_3_ NPs surrounding the A549 cells. **b** Elemental analysis of F atoms in RE-NPs distributed around the cell. **c** Elemental analysis of La atoms. The low concentration of La atoms was associated with a rather small scattering efficiency of the X-rays. **d**–**f** The same as for (**a**–**c**) for PrF_3_ RE-NPs

The TEM images and the elemental mapping of F, La and Pr showed that RE-NPs were unable to penetrate inside the cell. They gathered around the A549 cell membrane (Fig. 11), confirming that an external force can stimulate cell growth because of TSRs activation [44]. The Pr atoms were distributed around the boundaries of the cell’s membrane. The small numbers of F, La and Pr identifications inside the cell were not associated with endocytosis of RE-NPs, but they were images of RE-NPs from the projections of the two cells hemispheres on cell’s equatorial cycle.

It was also evident that both RE-NPs were able to enhance AKT phosphorylation, especially in A549 cells (Fig. 9d), where the steady-state level of AKT pathway activity was higher for the SW837 cell line. The phosphorylation level for the MCF7 cell line was below the detection limit, in agreement with the relatively low levels of growth. High phosphorylation levels of ERK1/2 [36] and AKT were detected in A549 and SW837 cell lines. Cell growth was started once NPs with a proper size interact with the mechanosensors of the cells to provide the correct force for initiating cell growth [45, 46]. ERK and AKT pathways were frequently active in several cancer cell types via extracellular springing, as they were stimulated by the TSRs, upon a selective binding with various mitogenic ligands, or via the activation of the mechanosensory group. The interaction was responsible for a continuous intracellular stimulation that, according to the cell’s phenotype, driven the cancer cells to uncontrolled and endless growth. Viability tests were also run for 48 and 72 h, but the growth of all cell lines was saturated at 48 and 72 h after the initial moment of Cell plating.

#### Interaction of Cells with Ions

Likewise, as fluoride anions are the most reactive electronegative elements and, the mean radii extension of the unscreened *4f* electronic configuration of La and Pr trivalent ions are relatively large, high electric surface charges could be developed via electric dipole interactions [47].

One crucial question stands whether a single ion binding on a specific site can activate tumour cell growth. Because the projected area of the *4f* electronic configuration of a single RE ion is *S*_*4f*_ = 0.040 and 0.043 nm^2^ (for an approximated spherical geometry of the *4f* electronic configuration and a *4f* mean orbital radii ~ *r*_4*f*_=0.113 and 0.117 nm for Pr and La ions, respectively), a typical upper limit number of single RE ions, or other equivalent size ions, over the whole area of the cell membrane was ~ S_c_/ *S*_*4f*_ = *N*_*4f*_ ~7.9 × 10^9^ RE ions; a number which is at least two orders of magnitude above the upper limit of the mean number of integrins on a tumour cell. As the relative overgrowth of cells was ascending with rising concentration (Fig. 9a), it is unlikely that tumour cell growth is triggered by a specific binding of single trivalent RE ions [48] on the ligand sites [49–51]. Indeed, the large number of RE ions should have saturated the cell’s growth and thus the viability of cells should have remain independent from the concentration of the RE ions.

#### Interaction of Integrins with RE-NPs

Within the requisite force range of few pN, and for efficient activation of integrins from NPs, the interaction between NPs and LABS should activate a large fraction of integrins of the cell for a long time. In the most extreme favoured case for cell growth, the number of NPs had to remain equal with the number of integrins on the cell’s surface, and the interactive force between LABS and NPs has to be attractive for obtaining a constant (long-term) action. A thin spherical shell of spherical NPs surrounding a tumour cell occupied a volume${V}_{sc}\approx 4\uppi {R}_c^2x$, where *R*_*c*_ = 5 μm is the cell radius and *x* ≈ 20 nm is half the separating distance between adjacent integrin receptors and *V*_*sc*_ ≈ 6.3 x 10^9^ nm^3^. For justifying the requirement that each integrin receptor interacts only with one NP, a first estimation of the size of NPs to meet the above requirements for the whole set of integrins on a cell is obtained by dividing the volume of the spherical shell *V*_*sc*_ with the number of integrins. A simple calculation for a cell radius 5 μm shows that the limits of radii of NPs activating the whole set of integrins within the spherical shell volume *V*_*sc*_ ≈ 6.3 x 10^9^ nm^3^ covering the cell is obtained by divided the volume *V*_*sc*_ with the number of integrins ${\overline{N}}_{int}\approx 2\ \mathrm{x}\ {10}^5$ and *n*_*int*_ ≈ 1.6 x 10^7^. The volume of the spherical NPs stands for 3.15 × 10^4^ and 3.93 × 10^2^ nm^3^ respectively. Therefore, the radii of the NPs interacting with an integrin lay between ~ 20 and 5 nm. Allowing for one order of magnitude variations in the number of integrins ${\overline{N}}_{int}$, the radii of the NPs interacting with integrins is between ~ 27 and ~  3 nm respectively.

By also applying similar simple calculations and within the experimental limits of concentration levels of RE-NPs (0.1–10 kg m^−3^), the *maximum* numbers of PrF_3_ with MHR 55–83 nm and LaF_3_ with MHR 296–100 nm NPs (Fig. 1g, h) covering the surface of a tumour cell *V*_*sc*_ stood for 4.1 × 10^4^–2.1 × 10^4^ and 17.1 × 10^2^–1.5 × 10^4^ NPs. These values are placed well below the number of integrins on the cell surface. For rising concentrations of PrF_3_ and LaF_3_ from 0.1 and 10 kg m^−3^, the number of PrF_3_ and LaF_3_ NPs in the suspensions must go up for either descending or ascending size of NPs. As viabilities of cancer cells are raised at higher concentration levels, it is unlikely that 55–296 nm sized RE-NPs are responsible for cancer cell mitosis under the current experimental configuration.

Also, from the DLS data, the size of both RE-NPs between 10 and 20 nm remained constant (10.6 nm) at different RE concentrations. The number of RE-NPs with this size covering the cell surface is between 3.7 × 10^5^ and 1.5 × 10^6^. This number is comparable with the mean number of integrins ${\overline{N}}_{int}\approx 2\ \mathrm{x}\ {10}^5$ on a cell surface. Therefore, only small size RE-NPs have the potency to stimulate cancer cell growth by stimulating all the integrins on a cell surface, in agreement with the experimental observations (Figs. 1g, h and 9a).

The number of tiny sizes RE-NPs with MEAC diameter (TEM) from 2 to 10 and 10 to 15 nm on the cell surface (*S*_*c*_ = 314 μm^2^) stands for 1.3 × 10^4^ and 1.8 × 10^4^ RE-NPs, respectively. Those values stayed one order of magnitude below ${\overline{N}}_{int}\approx 2\ \mathrm{x}\ {10}^5$ and therefore tiny size RE-NPs had also the potency to justify the experimental results of rising viability values with concentration (Fig. 9a). Also, the rough surface of tumour cell (Fig. 9c) is able to form cavities, where small size RE-NPs are trapped, triggering thus cell’s mechanosensors. Most important, only tiny size RE-NPs have the potency to activate integrin receptors via electrical dipole interactions (vide infra).

#### Interaction of EGFR with RE-NPs

**Fig. 12:**
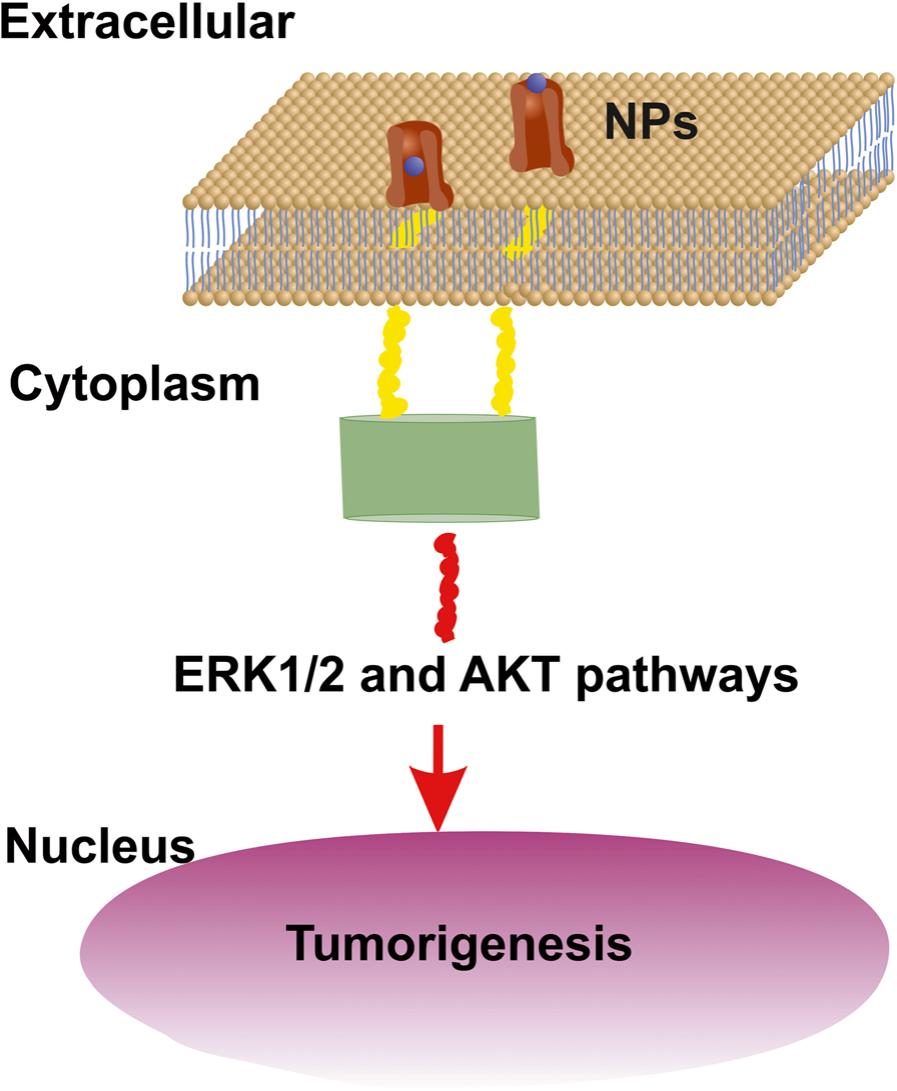
AKT and ERK1/2 signal transduction pathways activated by RE-NPs via EGFR stimulation. EGFR is activated only by tiny size ~ 5 nm NPs

An upper limit of small size NPs capable of stimulating cell’s overgrowth via the EGFR was set previously to 14 nm [52], but a realistic size of NPs stimulating the EGFR should be < 5 nm [53] (Fig. 12). The area number density of EGFR on the surface of tumour cells stands for ~ 1.4 × 10^−4^ nm^−2^ and the total number of EGFR on the surface *S*_*c*_ of cells remains between ~ 4.2 x 10^4^ and 10^5^ [54–56]. RE-NPs with 5–10 nm size stayed for a number of 34 NPs (Fig. 3). Extrapolating this number to the surface of a cell *S*_*c*_, the total number of RE-NPs remained at ~ 10^4^ NPs, a number which matches the number of EGFR receptors on a A549 cell. Therefore, the EGFR have the potency to be activated synergistically also by a number of tiny size RE-NPs.

### Electric Dipole Interaction Between RE-NPs and LABS

The above experimental results are supported by the hypothesis of cancer cell growth from LABS stimulation by tiny size core-shell RE-NPs via electrical dipole interactions, Appendix.

**Fig. 13:**
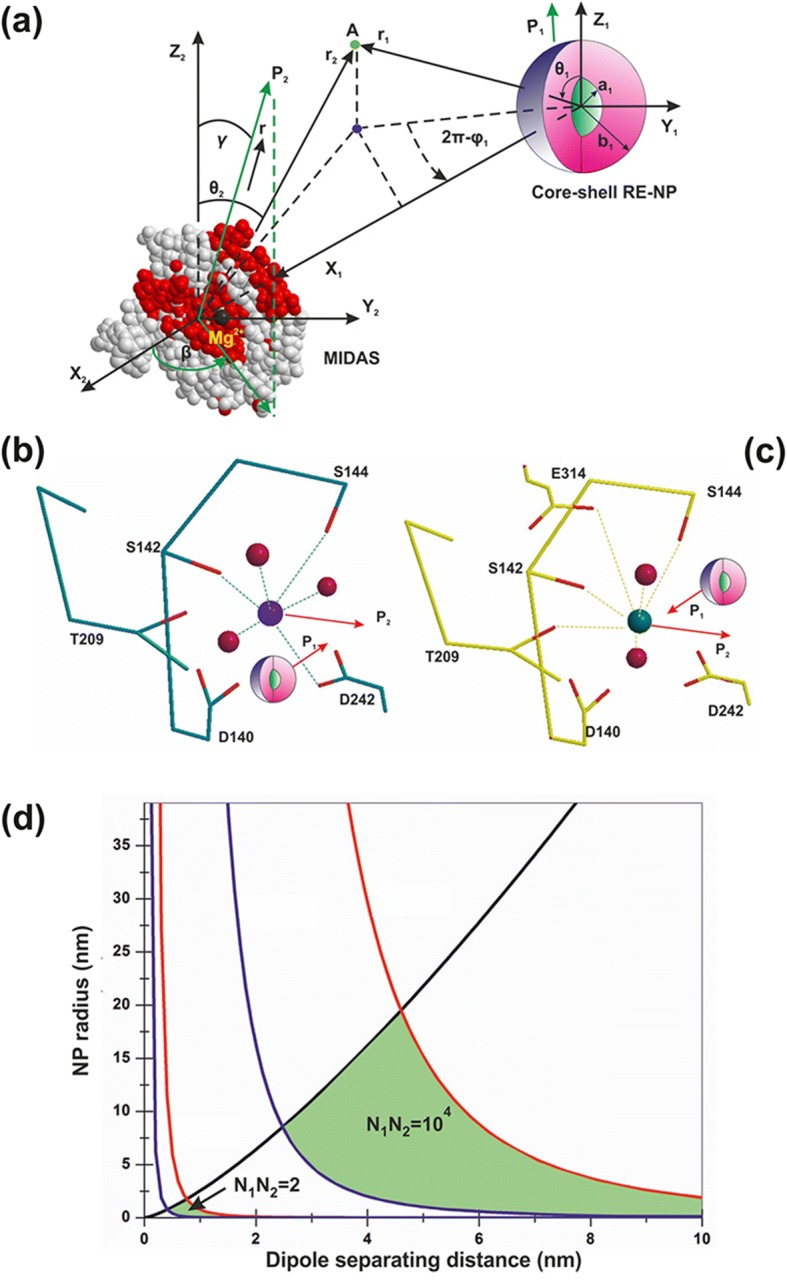
**a** Electrical dipole interaction between one core-shell RE-NP and one LABS. **b**, **c** RE core-shell NP near a MIDAS (**b**) and ADMIDAS (**c**) adhesion sites. **d** Locus area (green) of the size of RE-NPs and separating distance between a LABS and a core-shell RE-NP for two electrical charging states

Indeed, the mean electrical dipole force $\left\langle {\overrightarrow{F}}_{V_2}\right\rangle$acting on LABS from a core-shell RE-NP includes two terms (Fig. 13d and Appendix, Eq. A22). The first radial term is inversely proportional to the forth power of separating distance *r*_1_ between the RE-NPs and LABS and is also proportional to the size of NP. The second polar term is inversely proportional to both the separating distance *r*_1_ and the square power of the size of NP,

$$ \left\langle {\overrightarrow{F}}_{V_2}\right\rangle =-\frac{G_1{N}_2{N}_1\ d{e}^2}{4{\varepsilon}_0\ {r}_1\ }\theta \left(\ 3G\frac{b}{r_1^3}{\widehat{r}}_1+\frac{\theta }{2{b}^2}{\widehat{\theta}}_1\right)\kern0.75em (1) $$

In Eq. 1, *G* and *G*_1_ are the geometrical factors of NPs, describing either core-shell or core spherical structures, Appendix, Eqs. A6 and A14; *N*_1_, *N*_2_ are the numbers of surface electrons on the a RE-NPs and LABS surfaces; *d* and *b* are the effective characteristic spatial extension of atomic orbitals of LABS, ~ 0.1 nm, and the radius of RE-NP; *e* and *ε*_*0*_ are the electron charge and the vacuum permittivity and $\theta =\frac{d}{r_1}<0.01\  rad$. Because the core of the RE-NPs is a crystalline semiconductive material, an inherent large number of surface and volume defective sites were accountable for a high density of pseudo-electron energy levels that allowed the electrons to move freely within the core volume [46]. Consequently, a core-shell structure had the potency to be highly polarised. Therefore, LABS can be activated efficiently by core-shell RE-NPs via electrical dipole interactions at close separating distances. The high polarised efficiency of the core nucleus was confirmed experimentally via the selective orientation of NPs along two distinct directions (Fig. 2(a4–d4) and Fig. 3(a4, b4)).

The polar interaction force is also proportional to the geometrical factor *G*_1_, Appendix, Eq. A14. Typical values of dielectric constants of the culture media, shell configuration and RE core components stand for *ε*_1_ = 78, *ε*_2_ = 10 and *ε*_3_ = 15. When the ratio of core-shell to core radii *b/a* sets within 1 and 50, the geometrical factors *G*, *G*_1_ retain almost constant values (*G* = 0.2, *G*_1_ = 0.01) and they are self-same for both a spherical core (*b/a* = 1) and a spherical core-shell. Any permanent or induced polarisation of an open or closed a-I-MIDAS domain forming the LABS domain has its origin on six coordinated water oxygen atomic orbitals with Mn^2+^ or Mg^2+^ ions, arranged in a spherical geometric configuration [7] (Fig. 12a–c).

As the electrical dipole force in Eq. 1 stands for the vector sum of a radial (first term) and a polar component (second term), the last term prevails over the first one provided that

$$ {r}_1>\sqrt[3]{6G}b\sim b\kern0.75em (2) $$

In this case, a LABS is activated from the polar force component for all (b/a) ratios and, most important this term is inversely proportional to the second power of the size of NPs, in agreement with the experimental results that only tiny or small size LaF_3_ NPs activated cancer cell proliferation.

The prevailed polar force term for different *r*_1_ and *b* values and for different *Ν*_1_, *Ν*_2_ charging states activating the LABS/MIDAS stay within the limits [57–60].

$$ {10}^{-12}N<\frac{G_1{N}_2{N}_1\ d{e}^2}{8{\varepsilon}_0\ {r}_1{b}^2\ }{\theta}^2<{10}^{-9}N\kern1em (3) $$

Inequality 3 relates the size *b* of the RE core-shell NPs, the separating distance *r*_1_ and the number of the bound or free electrons *Ν*_2_, *Ν*_1_ on the surface of the two dipoles. The locus of points (*r*_1_, *b*) satisfying the inequality 3 for different surface charge states *Ν*_1_, *Ν*_2_ is bounded by the black, red and blue lines (Fig. 13c). As there was no specific assumptions for the type of RE-NP, results can be equally applied for any type of polarised NPs.

When the algebraic product of the number of the surface electrons *N*_1_ and *N*_2_ (bound or free) on the LABS and the RE-NP, respectively, was *N*_1_*N*_2_ = 2, the locus of RE-NPs size and separating distance for integrin activation was < 1 nm. At higher charging states, *N*_1_*N*_2_ = 10^4^, the locus area spans a wider RE-NPs size and separating distance area set of values, from 0.5 nm–19 nm to 2.5–15 nm, respectively.

From the above analysis, it is found that only tiny or small size NPs can activate LABS at a certain separating distance *r*_1_ and the electrical dipole interaction strength decays inversely proportional to the second power of the size of NPs. From Fig. 13c and for a charging state with *N*_1_ *N*_2_ = 5 x 10^4^, the size of NPs capable to activate LABS is bounded by the limits

$$ 2.5\ \mathrm{nm}<b<19\ \mathrm{nm} $$

Most important, from Fig. 13c, both the locus area (green area) and the size of RE-NPs increase for higher electrical charging states.

## Conclusions

Cancer is a complex disease. Tumours are highly heterogeneous, and cell growth, among other factors, depends on dynamical interactions between cells and the continually changing extracellular matrix. Besides random genomic mutations, signal transductions in cells, activating cell growth can be triggered by mechanical, thermodynamic and electrical polar interactions between the microenvironment of the extracellular cell matrix and the membrane’s mechanosensors. Here, we demonstrated that tumour cell proliferation in three different human cancer cell lines (A549, SW837, MCF7) had the potency to be activated by a synchronised and synergetic activation of EGFR or via electrical dipole interactions between tiny size RE-NPs and the LABS of integrins on a cell.

Because the prerequisite force for integrin activation should stand between 10^−12^ and 10^−9^ N, the size of the active RE-NPs causing cell growth should be within certain limits. Cancer activation is specified by both the electrical surface charges on the LABS and the NPs and by their separating distance. This electric dipole activating force follows an inversely proportional square power law of the radius of NPs, evidencing that only tiny or small size RE-NPs have the potency to stimulate cancer cell growth via electrical dipole interactions, in agreement with the experimental results.

## Methods

### Synthesis of RE-NPs

PrF_3_ NPs were synthesised via co-precipitation. Briefly, 4 g of Pr_2_O_3_ were added to 110 mL of 10% nitric acid in a polypropylene glass beaker together with 3 g of NaF under stirring. The mixture was heated to 50 °С and stirred for 45 min until a clear light-green solution appeared. Then it was filtered. The pH of the mixture adjusted to 4 by adding 25% of ammonium hydrate. Next, the mixture was stirred again for 20 min. Finally, the precipitated NPs washed with distilled water by centrifugation.

LaF_3_ NPs were also synthesised by applying the same protocol in a mixture of La_2_O_3_ (4 g) and NaF (3 g). From both preparations, an aliquot of the suspensions containing NPs was air-dried for structural analysis and the remaining part kept as water suspension for the biological studies.

The suspensions of NPs were prepared in complete DMEM+FBS cell culture medium by adding water suspended NPs directly to the medium to a final concentration of 5 mM. Then, starting from the 5 mM stock solution, some subsequent dilutions using DMEM as a solvent were prepared to a final NPs concentration of 1 mM and 0.5 mM, respectively.

### Size Distribution of RE-NPs

#### XRD

The crystal structure and the size of PrF_3_ and LaF_3_ NPs were characterised by XRD spectroscopy, with an X-ray diffractometer (Shimadzu XRD-7000S) in the 2θ range from 10° to 80° using the graphite monochromatised Cu-Ka radiation (1.5406 Å). The weighted average of τ for all peaks was used in the statistics. Weighting, besides β, took into account the relative intensity of every peak of the XRD spectra. The corresponding errors incorporate the reading error (0.3 mrad) and the standard error of the mean (*se* = *σ*/ √ *Ν*).

#### DLS

The size distribution and the MHR of RE-NPs in water and DMEM+FBS suspension were determined for comparison by DLS at 632.8 nm and right angles at 37 °C with a multi-angle dynamic and static light scattering instrument (PHOTOCOR-FC). The values of the MHR (Stokes radius) and the size distribution of NPs were calculated from the autocorrelation spectra and the Stokes-Einstein relation with the DynaLS software. Because the intensity of scattered light in pure DMEM+FBS was 20 times lower than with RE-NPs additives, the level of aggregating proteins in pure DMEM+FBS was negligible compared with mixed suspensions of RE-NPs in DMEM+FBS medium. MHR and RE-NPs size distribution and size errors were obtained by fitting and processing the data from the DLS instrument with the DynaLS software that allows the MHR to be calculated in different spectral domains of the main size distributions, from 10–10^2^ to 10^2^–10^3^ nm, Additional file 2.

#### AFM

Because size distribution below 15 nm was close to the low limit range of DLS, AFM was also applied to evaluate small size distribution. At low concentration of RE-NPs in liquid suspensions and slow drying rates of droplets on glass substrates, the deposits reflected the size distribution in the liquid suspensions [37]. Following the dispersion of RE-NPs in ethanol or DMEM+FBS, a drop of suspension was placed on a clean glass substrate using a micropipette, and then it was dried in air at room temperature for AFM imaging and analysis (diInnova, Bruker). AFM was performed in the tapping mode, in ambient conditions with a phosphorus-(n)-doped silicon cantilever (Bruker, RTESPA-CP), having a nominal spring constant of 40 nN/nm and operating at a resonance frequency of 300 kHz. Surface areas of various sizes (0.5 × 0.5–50 × 50 μm^2^) were imaged with high spatial resolution (512 px × 512 px) at a scanning rate of 0.2 Hz to identify domains with different size distributions via “scan area filtering” [37]. From the morphological analysis by the SPM LabAnalysis V7 software, the particle’s size distribution, shape and aggregation stage were determined.

The size of NPs for different scanning areas was also noticeable by the particle analysis chromatic bar (Fiji integrated ROI colour coder based on MEAC diameter) (Fig. 2(a1–d1)). The AFM image was transformed into a binary image using an appropriate *z*-height threshold. Every pixel of the processed image contained information not only for the *z*-height in the pixel area but also for the presence of particles in the pixel area. The *x*-histograms of MEAC and Ferret diameter (Fig. 2(a2–d2, a3–d3)) were extracted by using the “Image J 1.51n Fiji distribution software”, with the correct *z*-height threshold values. The size resolution per pixel was 3.9 and 1.9 nm for PrF_3_ and LaF_3_ respectively.

The particle identification, the noise extraction and the particle area data were processed by the “Particle Analyser function” of Fiji software (Fig. 2(a1–d1)). The particle diameter histograms were also analysed. Both the equal area circle diameter (Fig. 2(a2–d2)) and Feret diameter or “calliper diameter” (*maximum* diameter of a particle among all directions) (Fig. 2(a3–d3)), whose direction was the Feret angle (Fig. 2(a4–d4)), were analysed. The mean equal area circle diameter and the mean Feret diameter were calculated taking into account all particles identified. The associated errors incorporated the actual pixel size in every AFM image and the standard error of the mean (*se* = *σ*/ √ *Ν*).

A *t* test was performed for every set of AFM images based in the “null hypothesis” that the mean particle diameter was the same for all the AFM images between randomly selected figures (Fig. 2(a1–b1, c1, d1)). The *p* value (probability that the null hypothesis based on *t* distribution is not valid) is shown in Additional file 2.

#### TEM

The same technique was followed for calculating the above parameters in TEM imaging (Fig. 3(a1–b4)). Atomic resolution TEM (Hitachi HT7700 Exalens) imaged either extracellular or intracellular RE-NPs attachment on the A549 cells fixed in glutaraldehyde. Elemental analysis of F, La and Pr were also carried out (Oxford Instruments X-Max 80T).

#### 2D-FFT

Additional information on the NPs size distribution in the (*x*, *y*) plane was also extracted from the 2-D Fourier transform of AFM images of NPs using the relation

$$ I\left({k}_x,{k}_y\ \right)=\iint f\left(x,y\right)\exp \left(i{k}_xx\right)\exp \left(i{k}_yy\right) dxdy $$

where *f*(*x*, *y*) is a size function at a point (*x, y*), *k*_*x*_, *k*_*y*_ are the associated wavevectors in the inverse Eukledian space at the same point and *I*(*k*_*x*_, *k*_*y*_) is the “spectral density” of the function *f*(*x*, *y*) at the point *k*_*x*_, *k*_*y*_. For most applications, *f*(*x*, *y*) is the *z*-height of the NPs at the point (*x, y*) and *z* = *f*(*x*, *y*).

For a set of discrete data, such as the digitised AFM images, the 2D-FFT was used instead of 2D Fourier transform in the continuous space. For a *m* × *n* X-matrix (pixels of an AFM image), the 2D-FFT transform takes the form

$$ \kern1em {Y}_{p+1,q+1}=\sum \limits_{j=0}^{m-1}\sum \limits_{k=0}^{n-1}{\omega}_m^{jp}{\omega}_n^{kq}{X}_{j+1,k+1\kern1.25em } $$

where ${\omega}_m^{jp}={e}^{2 pi/m},{\omega}_n^{kq}={e}^{2 pi/n}$ are the associated frequencies. Then, an appropriate shift along the *y*-axis was performed and the integers m, n, p, q, k were translated into lengths and inverse lengths respectively by a multiplication with the pixel’s size of the image.

### Water Trapping in RE-NPs

#### VUV Spectroscopy

To appraise the state of water in RE-NP’s complexes during the initial stage of suspension preparation, the adsorption of water molecules on the surface of the hygroscopic PrF_3_ NPs was identified with a laboratory-made VUV (110–180 nm) absorption spectrometer. It consists of a hydrogen lamp operating in a longitudinal stabilised discharge mode at 10 kV, a stainless steel vacuum chamber and a VUV monochromator (Acton VM502), equipped with a solar blind photomultiplier (Thorn EMI 9412 CsTe) and a laboratory-made data collection system. Thin layers of PrF_3_ NPs suspensions in water were prepared and dried on 1-mm-thick VUV-grade CaF_2_ substrates by applying the “drop-casting method”. Then, the CaF_2_ substrates were placed in the optical path between the hydrogen lamp and the VUV monochromator in a vacuum. The stainless steel 316 vacuum chamber was evacuated initially to 10^− 7^ mbar using two turbomolecular pumps at a differential pumping configuration (Edwards EXT 100/200, pumping speed 150 ls^−1^). However, a high outgassing rate of PrF_3_ sets an upper limit to the background pressure in the vacuum chamber ~ 8.5 × 10^−5^ mbar. The relatively low background pressure of both compounds irreversibly damages the VUV optics and the turbomolecular pump after few hours of operation and therefore it sets certain experimental constraints, preventing an equivalent registration of LaF_3_ spectrum because of high outgassing rates and a low background operating pressure (< 10^−4^ mbar). The experimental data (light transmitted through the sample film on CaF_2_ window) were fitted to a logarithmic response for calculating the transmittance.

### Cell Culture and Growth Assay

#### Cell Growth

The A549 and SW837 cell lines were maintained in DMEM+FBS, whereas the MCF7 lines were in RPMI+FBS. Both media supplemented with 10% fetal bovine serum (FBS), 1 × penicillin, 1 × streptomycin and 2 mM l-glutamine. Cells were incubated at 37 °C, 5% CO_2_ in a humidified atmosphere.

The WST viability test was used to monitor the intrinsic toxicity of PrF_3_ and LaF_3_ NPs for three human cancer cell lines, A549, SW837 and MCF7. For the viability assay, three different concentrations of RE solubles (0.5, 1 and 5 mM) in DMEM+FBS (A549, SW837) and RPMI+FBS (MCF7) were used. The initial number of cells seeded in the 96-well plates was ~ 5 × 10^4^ cells/well. This amount of cells was plated 24 h prior to the RE-NPs treatment of cells in order to allow enough time for the cells to attach properly to the plate (wells) and to attain the optimum growing conditions. Subsequently, the viability test was performed 24 h after RE-NPs addition, or 48 h after the initial cell cultures were placed in the wells. As we did not observe any cell reduction, but on the contrary cell-overgrowth, especially with the SW620 cell line at 5 mM, the cell confluence quickly reached 80–90% of its initial value after 24 h of the addition of RE-NPs or 48 h from the initial plating.

Five microliters of WST solution was added to each well and the plate was incubated for 1 h during the growth state. The absorbance at 450 nm of each well was measured using a microplate reader (Biorad, x Mark). Each experimental point for each cell line and each RE suspension was extracted from two samples and triplicated every 2 days (total of 108 samples).

*F* test was used for every set of cell viability measurements. Here, the “null hypothesis” was that the relative to the CTRL “mean viability value was the same at different concentrations within the same cell line”. With this null hypothesis, an unknown law connecting tumour cell viability and RE-NPs concentration was identified. The *p* value (probability the null hypothesis to be rejected) was also tested from the *F* distribution Additional file 1.

#### Western Blotting and Antibodies

Total proteins were extracted with 60 μL of radioimmunoprecipitation assay (RIPA) lysis buffer (20 mM Tris-HCl (pH 7.5); 150 mM NaCl, 1 mM Na_2_EDTA; 1 mM EGTA; 1% NP-40; 1% sodium deoxycholate; 2.5 mM sodium pyrophosphate; supplemented with proteases inhibitors 1 mM β-glycerophosphate; 1 mM Na_3_VO_4_ 1 μg/ml; leupeptin) and the Wb assay was performed according to standard protocols (Fig. 9b). Briefly, total proteins (50 μg) were separated by SDS-polyacrylamide gel electrophoresis (SDS-PAGE) and transferred to nitrocellulose membrane. Blots were incubated overnight at 4 °C with appropriate primary antibodies. The antibodies used were tubulin code sc-8035, from Santa Cruz (final concentration 1:1000 in blocking buffer); p-ERK (E-4) code sc-7383, from Santa Cruz (final concentration 1:500 in blocking buffer); and p-AKT (Thr308) code 9275S, from Cell Signaling (final concentration 1:1000 in blocking buffer).

Wb bands are collected from different blots showing quality control of antibodies specificity. Numbers at the top of the phosphorylation images show grey scale levels from 0 (black) to 168 (grey) (*maximum value*), indicating activation at a non-saturated mode.

### Additional files

Additional file 1: Viability t- statistics of tumour cells. (DOCX 36 kb)

Additional file 2: NPs size t-statistics. (DOCX 2685 kb)

## Appendix

### Electrical Dipole Force Between One LABS and RE-NP

To describe the polar interaction between the LABS in integrins and the spherical core-shell dielectric RE-NPs the core-shell was placed at the origin *O*_1_ of an orthogonal Cartesian coordinate system with unit vectors $\left({\widehat{x}}_1,{\widehat{y}}_1,{\widehat{z}}_1\right)$ at a point A having an electric polarisation vector ${\overrightarrow{P}}_1$along the *z*_1_ axis with azimuthal symmetry around an angle *φ*_1_ on the (*x, y*) plane and a polar angle *θ*_1_ between the position vector ${\overrightarrow{r}}_1$ and the *z*_1_*-*axis, Fig. 13a. For simplicity, and without any loss of generality, the axes *z*_1_, *z*_2_ were set parallel. The polarisation vectors ${\overrightarrow{P}}_1,{\overrightarrow{P}}_2$are skew vectors for a permanent polarisation ${\overrightarrow{P}}_2$; otherwise, they are coplanar. An external stray electric field ${\overrightarrow{E}}_1\kern0.5em$ is derived from a scalar potential$\kern0.5em {\overrightarrow{E}}_1=-\nabla \varPhi$, satisfying certain boundary conditions at infinity and at the interface surfaces of the core and the shell accordingly. The electric potential derived from the external electric field can be expanded in a power series sum of Legendre polynomials *P*_*l*_(cos*θ*_1_)of *l* order at a space point *A*(*r*_1_, *φ*_1_, *θ*_1_) either outside (*r*_1_ > *b*) or inside (*r*_1_ < *b*) the core-shell.

The electric potential at the three distinct regions take the form,

$$ \kern0.5em {r}_1>b\kern1.75em {\varPhi}_1=-\varPhi \left(\infty \right)+\sum \limits_{l=0}^{\infty}\left(\frac{A_l}{r_1^{l+1}}\right){P}_l\left(\mathit{\cos}{\theta}_1\right) $$

$$ \kern3.00em a<{r}_1<b\kern1.5em {\varPhi}_2=\sum \limits_{l=0}^{\infty}\left({B}_l{r}_1^l+\frac{\varGamma_l}{r_1^{l+1}}\right){P}_l\left(\mathit{\cos}{\theta}_1\right)\kern3.75em \left(\mathrm{A}1\right) $$

$$ {r}_1<a\kern1.5em {\varPhi}_3=\sum \limits_{l=0}^{\infty }{\varDelta}_l{r}_1^l{P}_l\left(\mathit{\cos}{\theta}_1\right) $$

where *Φ*(∞) is the electric potential at infinity, *a, b* are the radius of the core and the shell respectively, ${\widehat{r}}_1,{\widehat{\theta}}_1$ are the radial and polar unit vectors in spherical coordinates. The continuity of the parallel (${\overrightarrow{n}}_1$) and perpendicular (${\overrightarrow{n}}_2$) components of the electric field ${\overrightarrow{E}}_1$ and the electric displacements vectors ${\overrightarrow{D}}_1,{\overrightarrow{D}}_2$ at the inner and outer surfaces of the core-shell, $\kern0.5em {\overrightarrow{n}}_1\bullet \left({\overrightarrow{E}}_1-{\overrightarrow{E}}_2\right)=0$ and ${\overrightarrow{n}}_2\bullet \left({\overrightarrow{D}}_1-{\overrightarrow{D}}_2\right)={\sigma}_f\left({\theta}_1,b\right)$, where *σ*_*f*_(*θ*_1_, *b*) is the surface free density of electric charges, ensure that only some of the *A*_*l*_, *B*_*l*_, *Γ*_*l*_, *Δ*_*l*_ coefficients survive. For a non-permanent polarisation, *σ*_*f*_(*θ*_1_, *b*) = 0.

For adapting universal boundary conditions at infinity, an isolated core-shell NP can be polarised under the action of a low external stray electric field ${\overrightarrow{E}}_0$, which is either zero or constant at infinity. In the case of a constant electric field, the potential at infinity *Φ*(∞) =  − *E*_0_*r*_1_ *cos θ*_1_. The boundary conditions at the two sides of the surfaces between the shell and the biological medium and between the shell and the core are described by the equations,

$$ {r}_1=b\kern1.75em {\varPhi}_1={\varPhi}_2,\kern0.75em {\varepsilon}_1\frac{\partial {\varPhi}_1}{\partial {r}_1}={\varepsilon}_2\frac{\partial {\varPhi}_2}{\partial {r}_1}\kern1.75em \left(\mathrm{A}2\right) $$

$$ {r}_1=a\kern1.75em {\varPhi}_2={\varPhi}_3,\kern0.75em {\varepsilon}_2\frac{\partial {\varPhi}_1}{\partial {r}_1}={\varepsilon}_3\frac{\partial {\varPhi}_2}{\partial {r}_1}\kern1.75em \left(\mathrm{A}3\right) $$

where *ε*_*ι* _, *ι* = 1, 2, 3, are the relative dielectric constants of the biological medium, the shell and the core, respectively. Using the boundary conditions A2, the coefficients *A*_1_, *B*_1_, *Γ*_1_, *Δ*_1_ are calculated to be

$$ {A}_1=\frac{\left({\varepsilon}_2-{\varepsilon}_3\right)\left({\varepsilon}_1+2{\varepsilon}_2\right){a}^3-\left({\varepsilon}_2-{\varepsilon}_1\right)\left({\varepsilon}_3+2{\varepsilon}_2\right){b}^3}{2\left({\varepsilon}_2-{\varepsilon}_3\right)\left({\varepsilon}_2-{\varepsilon}_1\right){a}^3-\left({\varepsilon}_2+2{\varepsilon}_1\right)\left({\varepsilon}_3+2{\varepsilon}_2\right){b}^3}{E}_0{b}^3 $$

$$ {B}_1=\frac{3{\varepsilon}_1\left({\varepsilon}_3+2{\varepsilon}_2\right)}{2\left({\varepsilon}_2-{\varepsilon}_3\right)\left({\varepsilon}_2-{\varepsilon}_1\right){a}^3-\left({\varepsilon}_2+2{\varepsilon}_1\right)\left({\varepsilon}_3+2{\varepsilon}_2\right){b}^3}{E}_0{b}^3 $$

$$ {\varGamma}_1=\frac{3{\varepsilon}_1\left({\varepsilon}_2-{\varepsilon}_3\right)}{2\left({\varepsilon}_2-{\varepsilon}_3\right)\left({\varepsilon}_2-{\varepsilon}_1\right){a}^3-\left({\varepsilon}_2+2{\varepsilon}_1\right)\left({\varepsilon}_3+2{\varepsilon}_2\right){b}^3}{E}_0{b}^3{\alpha}^3 $$

$$ {\varDelta}_1=\frac{9{\varepsilon}_1{\varepsilon}_2}{2\left({\varepsilon}_2-{\varepsilon}_3\right)\left({\varepsilon}_2-{\varepsilon}_1\right){a}^3-\left({\varepsilon}_2+2{\varepsilon}_1\right)\left({\varepsilon}_3+2{\varepsilon}_2\right){b}^3}{E}_0{b}^3 $$

By applying the boundary conditions and after some algebra, the potential *Φ*_1_ outside the core-shell is given by the equation,

$$ {r}_1>b\kern1.75em {\varPhi}_1=-{E}_0{r}_1\mathit{\cos}{\theta}_1+G\left(a,b,{\varepsilon}_{\iota}\right)\frac{b^3}{r_1^2}{E}_0\ \mathit{\cos}{\theta}_1\kern2.25em \left(\mathrm{A}4\right) $$

where *G*(*a*, *b*, *ε*_*ι*_) is a geometrical factor, equal with A_1_, which is a function of the core and shell radius a, b and the dielectric constants *ε*_*ι*_.

$$ G\left(a,b,{\varepsilon}_{\iota}\right)=G=\frac{\left({\varepsilon}_2-{\varepsilon}_3\right)\left({\varepsilon}_1+2{\varepsilon}_2\right){a}^3-\left({\varepsilon}_2-{\varepsilon}_1\right)\left({\varepsilon}_3+2{\varepsilon}_2\right){b}^3}{2\left({\varepsilon}_2-{\varepsilon}_3\right)\left({\varepsilon}_2-{\varepsilon}_1\right){a}^3-\left({\varepsilon}_2+2{\varepsilon}_1\right)\left({\varepsilon}_3+2{\varepsilon}_2\right){b}^3}\kern1.25em \left(\mathrm{A}5\right) $$

For a solid homogeneous sphere, *a = b*, ε_2_ = ε_3_ and Eq. A5 takes the form

$$ G=\frac{\varepsilon_1-{\varepsilon}_3}{\varepsilon_3+2{\varepsilon}_1} $$

The geometrical factor G attains a positive value for a solid sphere and a negative value for a core-shell structure of NPs when *ε*_1_ > *ε*_3._

From equation A4 and by using polar coordinates, the electric field outside the cell is given by the relationship,

$$ {\overrightarrow{E}}_1={E}_0\left(-\mathit{\cos}{\theta}_1{\widehat{r}}_1+\mathit{\sin}{\theta}_1{\widehat{\theta}}_1\right)-\frac{G{b}^3}{r_1^3}\left(2\mathit{\cos}{\theta}_1{\widehat{r}}_1+\mathit{\sin}{\theta}_1{\widehat{\theta}}_1\right){E}_0\kern0.5em \left(\mathrm{A}6\right) $$

It is interesting to note that the electric field falls as $\frac{1}{r_1^3}$ and the core-shell for $\frac{b}{r_1}<1$ behaves like an electric dipole with a dipole moment modulated by a shape geometrical factor G of the core-shell.

Today, it is unknown in what way the LABS of integrins (e.g. MIDAS adhesive sites) respond to an external electric field ${\overrightarrow{E}}_0.$ However, this is not important as the characteristic way in which the various multipoles of a geometrical polarised structure interact with an electric field shows that dipole interactions are far more effective than multipole ones. The LABS is considered to be fixed in space and the polarisation vector ${\overrightarrow{P}}_2={P}_2\widehat{r}$is the sum of a permanent and the induced polarisation vectors, making the angles γ and β relative to the *z*_2_ and *x*_2_ axes of a spherical coordinate system $\left({\widehat{r}}_2,{\widehat{\theta}}_2,{\widehat{\varphi}}_2\right)$ fixed with the ligand adhesion site, Fig. 8. The polarisation vector ${\overrightarrow{P}}_2$ takes the form

$$ {\overrightarrow{P}}_2={P}_2\widehat{r}={P}_2\left( sin\gamma cos\beta {\widehat{\chi}}_2+ sin\gamma sin\beta {\widehat{y}}_2+ cos\beta {\widehat{z}}_2\right)\kern1.25em \left(\mathrm{A}7\right) $$

where $\widehat{r}= sin\gamma cos\beta {\widehat{\chi}}_2+ sin\gamma sin\beta {\widehat{y}}_2+ cos\beta {\widehat{z}}_2$ is the unit vector of the polarisation vector ${\overrightarrow{P}}_2$in the coordinate system $\left({\widehat{r}}_2,{\widehat{\theta}}_2,{\widehat{\varphi}}_2\right)$. The total force ${\overrightarrow{F}}_{12}$ exerted from the core-shell and the external field on the ligand adhesion site at a point A (${\widehat{\theta}}_2,{\widehat{r}}_2\Big)$ is the sum of the volume ${\left({\overrightarrow{F}}_{12}\right)}_{V_2}$and the surface ${\left({\overrightarrow{F}}_{12}\right)}_{s_2}$ force components

$$ \overrightarrow{F}={\left({\overrightarrow{F}}_{12}\right)}_{V_2}+{\left({\overrightarrow{F}}_{12}\right)}_{s_2}=\kern1em -{\int}_{V_2}\left({\overrightarrow{P}}_2\bullet {\nabla}_1\right){\overrightarrow{E}}_1d{V}_2+{\int}_{s_2}\left({\overrightarrow{P}}_2\bullet {\widehat{r}}_2\right){\overrightarrow{E}}_1d{s}_2\Big]\kern1em \left(\mathrm{A}8\right) $$

### Volume Force Term

First, the volume force term ${\overrightarrow{F}}_{12}$ is evaluated. The gradient operator *∇*_1_ is associated with the coordinate system of the core shell, A (${\widehat{\theta}}_1,{\widehat{r}}_1\Big)$ and the integration is taken over the volume *V*_2_ and the surface *S*_2_ of the ligand adhesion site. For the most general case, the two polarizations are skew vectors and the volume force term ${\left({\overrightarrow{F}}_{12}\right)}_{V_2}$is calculated for *φ*_2_ ∈ [0, 2*π*] and *θ*_2_ ∈ [0, *π*].

$$ {\left({\overrightarrow{F}}_{12}\right)}_{V_2}=-{\int}_{V_2}\left({\overrightarrow{P}}_2\bullet {\nabla}_1\right){\overrightarrow{E}}_1d{V}_2=-{P}_2{\int}_{V_2}\left(\widehat{r}\bullet {\nabla}_1\right){\overrightarrow{E}}_1d{V}_2=\frac{G{b}^3{d}^3{E}_0{P}_2}{3}{\int}_0^{2\pi }d{\varphi}_2{\int}_0^{\pi}\left(\left(\widehat{r}\bullet {\widehat{r}}_1\right)\frac{\partial }{\partial {r}_1}+\left(\widehat{r}\bullet {\widehat{\theta}}_1\right)\frac{1}{r_1}\frac{\partial }{\partial {\theta}_1}\right)\left[\frac{1}{r_1^3}\left(2\mathit{\cos}{\theta}_1{\widehat{r}}_1+\mathit{\sin}{\theta}_1{\widehat{\theta}}_1\right)\mathit{\sin}{\theta}_2d{\theta}_2\right]-\frac{d^3{E}_0{P}_2}{3}{\int}_0^{2\pi }d{\varphi}_2{\int}_0^{\pi}\left(\left(\widehat{r}\bullet {\widehat{r}}_1\right)\frac{\partial }{\partial {r}_1}+\left(\widehat{r}\bullet {\widehat{\theta}}_1\right)\frac{1}{r_1}\frac{\partial }{\partial {\theta}_1}\right)\left(-\mathit{\cos}{\theta}_1{\widehat{r}}_1+\mathit{\sin}{\theta}_1{\widehat{\theta}}_1\right)\mathit{\sin}{\theta}_2d{\theta}_2=\frac{G{b}^3{d}^3{E}_0{P}_2}{3}{\int}_0^{2\pi }d{\varphi}_2{\int}_0^{\pi}\left(\left(\widehat{r}\bullet {\widehat{r}}_1\right)\frac{\partial }{\partial {r}_1}+\left(\widehat{r}\bullet {\widehat{\theta}}_1\right)\frac{1}{r_1}\frac{\partial }{\partial {\theta}_1}\right)\left[\frac{1}{r_1^3}\left(2\mathit{\cos}{\theta}_1{\widehat{r}}_1+\mathit{\sin}{\theta}_1{\widehat{\theta}}_1\right)\mathit{\sin}{\theta}_2d{\theta}_2\right]-\frac{d^3{E}_0{P}_2}{3}{\int}_0^{2\pi }d{\varphi}_2{\int}_0^{\pi}\mathit{\sin}{\theta}_2d{\theta}_2\left(\left(\widehat{r}\bullet {\widehat{r}}_1\right)\frac{\partial }{\partial {r}_1}+\left(\widehat{r}\bullet {\widehat{\theta}}_1\right)\frac{1}{r_1}\frac{\partial }{\partial {\theta}_1}\right)\left(-\mathit{\cos}{\theta}_1{\widehat{r}}_1+\mathit{\sin}{\theta}_1{\widehat{\theta}}_1\right)=-\frac{G{b}^3{d}^3{E}_0{P}_2}{r_1^4}{\int}_0^{2\pi }d{\varphi}_2{\int}_0^{\pi}\mathit{\sin}{\theta}_2d{\theta}_2\left(\widehat{r}\bullet {\widehat{r}}_1\right)\left(2\mathit{\cos}{\theta}_1{\widehat{r}}_1+\mathit{\sin}{\theta}_1{\widehat{\theta}}_1\right)-\frac{G{b}^3{d}^3{E}_0{P}_2}{3{r}_1^4}{\int}_0^{2\pi }d{\varphi}_2{\int}_0^{\pi}\mathit{\sin}{\theta}_2d{\theta}_2\ \left(\widehat{r}\bullet {\widehat{\theta}}_1\right)\left(2\mathit{\sin}{\theta}_1{\widehat{r}}_1-\mathit{\cos}{\theta}_1{\widehat{\theta}}_1\right)-\frac{d^3{E}_0{P}_2}{3{r}_1}{\int}_0^{2\pi }d{\varphi}_2{\int}_0^{\pi}\mathit{\sin}{\theta}_2d{\theta}_2\left(\widehat{r}\bullet {\widehat{\theta}}_1\right)\left(\mathit{\sin}{\theta}_1{\widehat{r}}_1+\mathit{\cos}{\theta}_1{\widehat{\theta}}_1\right)\kern1.25em \left(\mathrm{A}9\right) $$

Equation A9 is written as

$$ {\left({\overrightarrow{F}}_{12}\right)}_{V_2}=-\frac{d^3{E}_0{P}_2}{G^{1/3}\lambda\ \sqrt[3]{\lambda }\ b}{\int}_0^{2\pi }d{\varphi}_2{\int}_0^{\pi}\mathit{\sin}{\theta}_2d{\theta}_2\left\{\left[2\left(\widehat{r}\bullet {\widehat{r}}_1\right)\mathit{\cos}{\theta}_1+\frac{1}{3}\left(\lambda +2\right)\left(\widehat{r}\bullet {\widehat{\theta}}_1\right)\mathit{\sin}{\theta}_1\right]{\widehat{r}}_1+\left[\left(\widehat{r}\bullet {\widehat{r}}_1\right)\mathit{\sin}{\theta}_1+\frac{1}{3}\left(\lambda -1\right)\left(\widehat{r}\bullet {\widehat{\theta}}_1\right)\mathit{\cos}{\theta}_1\right]{\widehat{\theta}}_1\right\}\kern6.5em \left(\mathrm{A}10\right) $$

where λ is a dimensionless parameter of the ratio between the *r*_*1*_ and the size of the RE-NP *b* and the geometrical factor G

$$ \lambda =\frac{1}{G}{\left(\frac{r_1}{b}\right)}^3 $$

The electric potential, the electric field and the polarisation at the external boundary surface of the core-shell can be extracted by equating the bound surface charge density *σ*_*b*_(*θ*_1_, *b*) at the external surface of the core-shell NP with the component of the polarisation vector$\kern0.5em {\overrightarrow{P}}_1$. From Eq. A1, the normal components of the electric across the external shell surface are connected via the equations

$$ {\varepsilon}_1{E}_0={\varepsilon}_2{E}_2\Rightarrow {E}_2=-\frac{1}{\varepsilon_2}\ \nabla {\varPhi}_2= $$

$$ -\frac{1}{\varepsilon_2}\left({B}_1-\frac{2{\varGamma}_1}{b^3}\right)=-\frac{1}{\varepsilon_2}\left[\frac{3{\varepsilon}_1\left({\varepsilon}_3+2{\varepsilon}_2\right)}{2\left({\varepsilon}_2-{\varepsilon}_3\right)\left({\varepsilon}_2-{\varepsilon}_1\right){a}^3-\left({\varepsilon}_2+2{\varepsilon}_1\right)\left({\varepsilon}_3+2{\varepsilon}_2\right){b}^3}{E}_0{b}^3-\frac{2}{b^3}\frac{3{\varepsilon}_1\left({\varepsilon}_2-{\varepsilon}_3\right)}{2\left({\varepsilon}_2-{\varepsilon}_3\right)\left({\varepsilon}_2-{\varepsilon}_1\right){a}^3-\left({\varepsilon}_2+2{\varepsilon}_1\right)\left({\varepsilon}_3+2{\varepsilon}_2\right){b}^3}{E}_0{b}^3{\alpha}^3\right] $$

And finally

$$ {E}_2=\frac{2\left({\varepsilon}_2-{\varepsilon}_3\right){\alpha}^3-3{\varepsilon}_1\left({\varepsilon}_3+2{\varepsilon}_2\right){b}^3}{2{\varepsilon}_2\left({\varepsilon}_2-{\varepsilon}_3\right)\left({\varepsilon}_2-{\varepsilon}_1\right){a}^3-\left({\varepsilon}_2+2{\varepsilon}_1\right)\left({\varepsilon}_3+2{\varepsilon}_2\right){b}^3}{E}_0\kern2em \left(\mathrm{A}11\right) $$

For a solid sphere the last equation transforms to

$$ {E}_2=\frac{3{\varepsilon}_1}{\varepsilon_3+2{\varepsilon}_1}{E}_0 $$

The polarisation vector *P*_1_ inside the shell is related to *Ε*_2_ via the equation

$$ {P}_1={\varepsilon}_0\left({\varepsilon}_2-1\right){E}_2={\varepsilon}_0\left({\varepsilon}_2-1\right)\frac{2\left({\varepsilon}_2-{\varepsilon}_3\right){\alpha}^3-3{\varepsilon}_1\left({\varepsilon}_3+2{\varepsilon}_2\right){b}^3}{2{\varepsilon}_2\left({\varepsilon}_2-{\varepsilon}_3\right)\left({\varepsilon}_2-{\varepsilon}_1\right){a}^3-\left({\varepsilon}_2+2{\varepsilon}_1\right)\left({\varepsilon}_3+2{\varepsilon}_2\right){b}^3}{E}_0\kern0.75em \left(\mathrm{A}12\right) $$

and from Eq. A10, the external field *E*_0_ is related with the surface charge density *σ*_*b*_(*θ*_1_, *b*) at the surface of the shell at *b* and in the direction *θ*_1_ via the equation

$$ {E}_0={G}_1\frac{P_1}{\varepsilon_0}\kern0.75em \left(\mathrm{A}13\right) $$

From Eqs. A12 and A13, the geometrical factor *G*_1_ is

$$ {G}_1=\frac{1}{\left({\varepsilon}_2-1\right)}\frac{2{\varepsilon}_2\left({\varepsilon}_2-{\varepsilon}_3\right)\left({\varepsilon}_2-{\varepsilon}_1\right){a}^3-\left({\varepsilon}_2+2{\varepsilon}_1\right)\left({\varepsilon}_3+2{\varepsilon}_2\right){b}^3}{2\left({\varepsilon}_2-{\varepsilon}_3\right){\alpha}^3-3{\varepsilon}_1\left({\varepsilon}_3+2{\varepsilon}_2\right){b}^3}\kern1.5em \left(\mathrm{A}14\right) $$

The unit vectors ${\widehat{r}}_1$ and ${\widehat{r}}_2$ are associated with the two parallel coordinate systems. One unit vector is fixed with the coordinate system of the core-shell $\left({\widehat{r}}_1,{\widehat{\theta}}_1,{\widehat{\varphi}}_1\right)$and the other with the coordinate system of the ligand adhesion site, $\left({\widehat{r}}_2,{\widehat{\theta}}_2,{\widehat{\varphi}}_2\right).$ The components of ${\widehat{r}}_1$ and ${\widehat{r}}_2$ are connected with the Cartesian unit vectors via the relations

$$ {\widehat{r}}_1=\mathit{\sin}{\theta}_1\mathit{\cos}{\varphi}_1{\widehat{\chi}}_1+\mathit{\sin}{\theta}_1\mathit{\sin}{\varphi}_1{\widehat{y}}_1+\mathit{\cos}{\theta}_1{\widehat{z}}_1 $$

$$ {\widehat{r}}_2=\mathit{\sin}{\theta}_2\mathit{\cos}{\varphi}_2{\widehat{\chi}}_2+\mathit{\sin}{\theta}_2\mathit{\sin}{\varphi}_2{\widehat{y}}_2+\mathit{\cos}{\theta}_2{\widehat{z}}_2 $$

$$ {\widehat{\theta}}_1=\mathit{\cos}{\theta}_1\mathit{\cos}{\varphi}_1{\widehat{\chi}}_1+\mathit{\cos}{\theta}_1\mathit{\sin}{\varphi}_1{\widehat{y}}_1-\mathit{\sin}{\theta}_1{\widehat{z}}_1\kern1.5em \left(\mathrm{A}15\right) $$

$$ {\widehat{\theta}}_2=\mathit{\cos}{\theta}_2\mathit{\cos}{\varphi}_2{\widehat{\chi}}_2+\mathit{\cos}{\theta}_2\mathit{\sin}{\varphi}_2{\widehat{y}}_2-\mathit{\sin}{\theta}_2{\widehat{z}}_2 $$

$$ \widehat{r}= sin\gamma cos\beta {\widehat{\chi}}_2+ sin\gamma sin\beta {\widehat{y}}_2+ cos\gamma {\widehat{z}}_2 $$

The inner product of the unit vectors $\widehat{r}\bullet {\widehat{r}}_{1\kern0.5em },\kern0.5em \widehat{r}\bullet {\widehat{\theta}}_1$ and $\widehat{r}\bullet {\widehat{r}}_{2\kern0.5em },\kern0.5em \widehat{r}\bullet {\widehat{\theta}}_2$ are both functions of the polar and the azimuthal angles of the position and the polarisation vectors${\widehat{r}}_1,{\widehat{r}}_2$ and ${\overrightarrow{P}}_2$, Fig. 8. For ${\widehat{\chi}}_1\bullet {\widehat{\chi}}_2={\widehat{y}}_1\bullet {\widehat{y}}_2={\widehat{z}}_1\bullet {\widehat{z}}_2=1,$the product of unit vectors in spherical coordinated is

$$ \widehat{r}\bullet {\widehat{r}}_1= sin\gamma cos\beta sin{\theta}_1\mathit{\cos}{\varphi}_1+ sin\gamma sin\beta sin{\theta}_1\mathit{\sin}{\varphi}_1+ cos\gamma cos{\theta}_1 $$

$$ \widehat{r}\bullet {\widehat{\theta}}_1= sin\gamma cos\beta cos{\theta}_1\mathit{\cos}{\varphi}_1+ sin\gamma sin\beta cos{\theta}_1\mathit{\sin}{\varphi}_1- cos\gamma sin{\theta}_1\kern1.5em \left(\mathrm{A}16\right) $$

$$ \widehat{r}\bullet {\widehat{r}}_2= sin\gamma cos\beta sin{\theta}_2\mathit{\cos}{\varphi}_2+ sin\gamma sin\beta sin{\theta}_2\mathit{\sin}{\varphi}_2+ cos\gamma cos{\theta}_2 $$

$$ \widehat{r}\bullet {\widehat{\theta}}_2= sin\gamma cos\beta cos{\theta}_2\mathit{\cos}{\varphi}_2+ sin\gamma sin\beta cos{\theta}_2\mathit{\sin}{\varphi}_2- cos\gamma sin{\theta}_2 $$

By substituting Eqs. A15 to A10, the volume force term of the dipole interaction takes the form,

$$ {\left({\overrightarrow{F}}_{12}\right)}_{V_2}=-\frac{d^3{P}_2{E}_0}{G^{1/3}\lambda \sqrt[3]{\lambda }b}{\int}_0^{2\pi }d{\varphi}_2{\int}_0^{\pi}\sin {\theta}_2d{\theta}_2\left\{\left[2\left( sin\gamma cos\beta sin{\theta}_1\cos {\varphi}_1+ sin\gamma sin\beta sin{\theta}_1\sin {\varphi}_1+ cos\gamma cos{\theta}_1\right)\cos {\theta}_1+\frac{1}{3}\left(\lambda +2\right)\left( sin\gamma cos\beta cos{\theta}_1\cos {\varphi}_1+ sin\gamma sin\beta cos{\theta}_1\sin {\varphi}_1- cos\gamma sin{\theta}_1\right)\sin {\theta}_1\right]{\hat{r}}_1+\left[\left( sin\gamma cos\beta sin{\theta}_1\cos {\varphi}_1+ sin\gamma sin\beta sin{\theta}_1\sin {\varphi}_1+ cos\gamma cos{\theta}_1\right)\sin {\theta}_1+\frac{1}{3}\left(\lambda -1\right)\left( sin\gamma cos\beta cos{\theta}_1\cos {\varphi}_1+ sin\gamma sin\beta cos{\theta}_1\sin {\varphi}_1- cos\gamma sin{\theta}_1\right)\cos {\theta}_1\right]{\hat{\theta}}_1\right\}\kern8.25em \left(\mathrm{A}17\right) $$

To evaluate the total force $\overrightarrow{F}$ exerted on the LABS, Eq. A17 must be integrated for the variable *φ*_1_ ranging from 0^o^ to 2π and the variable *θ*_1_ from 0 to $\theta =\frac{d}{r_1}$, *θ* is the angle where the ligand adhesion site is projected from the center of the RE-NP.

$$ {\overrightarrow{F}}_{V_2}={\int}_0^{2\uppi}{\int}_0^{\theta }{\left({\overrightarrow{F}}_{12}\right)}_{V_2}{d\varphi}_1d{\theta}_1=-\frac{d^3{P}_2{E}_0}{G^{\frac{1}{3}}\lambda \sqrt[3]{\lambda }b}{\int}_0^{2\pi }{d\varphi}_1{\int}_0^{\theta }d{\theta}_1{\int}_0^{2\pi }d{\varphi}_2{\int}_0^{\pi}\sin {\theta}_2d{\theta}_2\left\{\left[2\left( sin\gamma cos\beta sin{\theta}_1\cos {\varphi}_1+ sin\gamma sin\beta sin{\theta}_1\sin {\varphi}_1+ cos\gamma cos{\theta}_1\right)\cos {\theta}_1+\frac{1}{3}\left(\lambda +2\right)\left( sin\gamma cos\beta cos{\theta}_1\cos {\varphi}_1+ sin\gamma sin\beta cos{\theta}_1\sin {\varphi}_1- cos\gamma sin{\theta}_1\right)\sin {\theta}_1\right]{\hat{r}}_1\right]+\left[\left( sin\gamma cos\beta sin{\theta}_1\cos {\varphi}_1+ sin\gamma sin\beta sin{\theta}_1\sin {\varphi}_1+ cos\gamma cos{\theta}_1\right)\sin {\theta}_1+\frac{1}{3}\left(\lambda -1\right)\left( sin\gamma cos\beta cos{\theta}_1\cos {\varphi}_1+ sin\gamma sin\beta cos{\theta}_1\sin {\varphi}_1- cos\gamma sin{\theta}_1\right)\cos {\theta}_1\right]{\hat{\theta}}_1\Big\}=\kern0.5em -\frac{4\pi {d}^3{P}_2{E}_0}{G^{\frac{1}{3}}\lambda \sqrt[3]{\lambda }b} cos\gamma \left\{{\int}_0^{\theta } d\theta \left(\left(2{\cos}^2{\theta}_1+\frac{1}{3}\left(\lambda +2\right){\sin}^2{\theta}_1\right){\hat{r}}_1+\frac{1}{3}\left(\lambda +2\right)\cos {\theta}_1\sin {\theta}_1{\hat{\theta}}_1\right)\right\}=-\frac{4\pi {d}^3{P}_2{E}_0}{G^{\frac{1}{3}}\lambda \sqrt[3]{\lambda }b} cos\gamma \left\{{\int}_0^{\theta } d\theta \left(2{\cos}^2{\theta}_1+\frac{2}{3}{\sin}^2{\theta}_1\right){\hat{r}}_1+\frac{2}{3}\cos {\theta}_1\sin {\theta}_1{\hat{\theta}}_1\Big)\right\}-\frac{4\pi {d}^3{P}_2{E}_0}{3{G}^{\frac{1}{3}}\sqrt[3]{\lambda }b} cos\gamma \left\{{\int}_0^{\theta } d\theta \left({\sin}^2{\theta}_1{\hat{r}}_1+\cos {\theta}_1\sin {\theta}_1{\hat{\theta}}_1\right)\right\}\kern0.75em \left(\mathrm{A}18\right) $$

After integration, Eq. A18 becomes,

$$ {\overrightarrow{F}}_{V_2}=-\frac{4\pi {d}^3{P}_2{E}_0}{G^{\frac{1}{3}}\lambda\ \sqrt[3]{\lambda }\ b}\kern0.5em cos\gamma \left[\left(4\theta +\mathit{\sin}2\theta \right){\widehat{r}}_1-2{\mathit{\sin}}^2\theta {\widehat{\theta}}_1\right]-\frac{4\pi {d}^3{P}_2{E}_0}{3{G}^{\frac{1}{3}}\ \sqrt[3]{\lambda }\ b} cos\gamma \left[\left(\frac{\theta }{2}-\frac{\mathit{\sin}2\theta }{4}\right){\widehat{r}}_1-{\mathit{\sin}}^2\theta {\widehat{\theta}}_1\right]\ \left(\mathrm{A}19\right) $$

Because $\frac{d}{r_1}$is small θ~sinθ and Eq. A19 read as

$$ {\overrightarrow{F}}_{V_2}\approx -\frac{24\pi {d}^3{P}_2{E}_0}{G^{\frac{1}{3}}\lambda \sqrt[3]{\lambda }b}\theta cos\gamma {\hat{r}}_1+\left(\frac{8\pi {d}^3{P}_2{E}_0}{G^{\frac{1}{3}}\lambda \sqrt[3]{\lambda }b}\kern0.5em +\frac{4\pi {d}^3{P}_2{E}_0}{3{G}^{\frac{1}{3}}\sqrt[3]{\lambda }b}\right){\theta}^2 cos\gamma {\hat{\theta}}_1=-\frac{4\pi {d}^3{P}_2{E}_0}{G^{\frac{1}{3}}\lambda \sqrt[3]{\lambda }b}\theta cos\gamma \left(6{\hat{r}}_1+\left(2+\lambda \right)\theta {\hat{\theta}}_1\right)=-\frac{4\pi {Gd}^3{P}_2{b}^3{E}_0}{r_1^4}\theta cos\gamma \left(6{\hat{r}}_1+\left(2+\frac{1}{G}{\left(\frac{r_1}{b}\right)}^3\right)\theta {\hat{\theta}}_1\right)\kern1.25em \left(\mathrm{A}20\right) $$

The polarizations *P*_1, 2_ are defined as the mean bound surface charge *Q*_1,2*b*_ = *N*_1,2_*e* divided by the surface. *N*_*1,2*_ is the number of surface bound electrons on the surface of the core shell and the LABS, respectively. By using Eqs. A13 and A20 read as

$$ {\overrightarrow{F}}_{V_2}=-\frac{G{G}_1{N}_2{N}_1 db{e}^2}{4\pi {\varepsilon}_0{r}_1^4}\theta cos\gamma \left(6{\hat{r}}_1+\left(2+\frac{1}{G}{\left(\frac{r_1}{b}\right)}^3\right)\theta {\hat{\theta}}_1\right)=-\frac{G{G}_1{N}_2{N}_1 db{e}^2}{4\pi {\varepsilon}_0{r}_1^4}\theta cos\gamma \left(6{\hat{r}}_1+2\theta {\hat{\theta}}_1\right)-\frac{G_1{N}_2{N}_1d{e}^2}{4\pi {\varepsilon}_0{r}_1{b}^2}{\theta}^2 cos\gamma {\hat{\theta}}_1\kern0.75em \left(\mathrm{A}21\right) $$

Averaging the last equation over the angle γ, we arrive at the final equation for the interaction force between LABS and RE-NPs,

$$ \left\langle {\overrightarrow{F}}_{V_2}\right\rangle =-\frac{G{G}_1{N}_2{N}_1 db{e}^2}{8{\varepsilon}_0{r}_1^4}\theta \left(6{\hat{r}}_1+\left(2+\frac{1}{G}{\left(\frac{r_1}{b}\right)}^3\right)\theta {\hat{\theta}}_1\right)\approx -\frac{G{G}_1{N}_2{N}_1 db{e}^2}{8{\varepsilon}_0{r}_1^4}\theta \left(6{\hat{r}}_1+2\theta {\hat{\theta}}_1\right)-\frac{G_1{N}_2{N}_1d{e}^2}{8{\varepsilon}_0{r}_1{b}^2}{\theta}^2{\hat{\theta}}_1\approx -\frac{3G{G}_1{N}_2{N}_1 db{e}^2}{4{\varepsilon}_0{r}_1^4}\theta {\hat{r}}_1-\frac{G_1{N}_2{N}_1d{e}^2}{8{\varepsilon}_0{r}_1{b}^2}{\theta}^2{\hat{\theta}}_1=-\frac{G_1{N}_2{N}_1d{e}^2}{4{\varepsilon}_0{r}_1}\theta \left(3G\frac{b}{r_1^3}{\hat{r}}_1+\frac{\theta }{2{b}^2}{\hat{\theta}}_1\right)\kern0.5em \left(\mathrm{A}22\right) $$

### Surface Force Term

The surface force term is calculated for *θ*_2_ ∈ [0, *π*].

$$ {\left({\overrightarrow{F}}_{12}\right)}_{s_2}={\int}_{s_2}\left({\overrightarrow{P}}_2\bullet {\widehat{r}}_2\right){\overrightarrow{E}}_1d{s}_2={P}_2{d}^2{\overrightarrow{E}}_1{\int}_0^{2\pi }d{\varphi}_2{\int}_0^{\pi}\left( sin\gamma cos\beta sin{\theta}_2\mathit{\cos}{\varphi}_2+ sin\gamma sin\beta sin{\theta}_2\mathit{\sin}{\varphi}_2+ cos\gamma cos{\theta}_2\right)\mathit{\sin}{\theta}_2d{\theta}_2=0\kern0.75em (A23) $$

## Abbreviations

2D-FFT: Two-dimensional fast Fourier transform
ADMIDAS: Adjacent MIDAS
AFM: Atomic force microscopy
AKT: Protein kinase B
CTRL: Control cells
DLS: Dynamic light scattering
DMEM: Dulbecco’s modified Eagle’s medium
ECM: Cell-extracellular matrix
EGFR: Epidermal growth factor receptors
ERK: Extracellular signal-regulated kinase
F.A.: Feret angle
F.D.: Feret area diameters
FBS: Fetal bovine serum
LABS: Ligand adhesion binding site
MEAC: Mean equal area circle
MHR: Mean hydrodynamic radius
MIDAS: Metal ion-dependent adhesion sites
NGFR: Nerve growth factor receptor
NP: Nanoparticle
RE-NPs: Rare-earth nanoparticles
RIPA: Radioimmunoprecipitation assay
RMS: Root mean square
RPMI: Roswell Park Memorial Institute medium
SDS-PAGE: Sodium dodecyl sulfate-polyacrylamide gel electrophoresis
SyMBS: Synergistic metal ion binding sites
TEM: Transmission electron microscopy
TSR: Transmembrane signal receptors
VEGFR: Vascular endothelial growth factor
VUV: Vacuum ultraviolet
Wb: Western blot assays
WST: Water-soluble tetrazolium salts
XRD: X-ray diffraction
