# Supplementary material for: Tiny Rare-Earth Fluoride Nanoparticles Activate Tumour Cell Growth via Electrical Polar Interactions
Source: Nanoscale Res Lett. 2018 Nov 21;13:370. doi: 10.1186/s11671-018-2775-z (PMC6249154; doi:10.1186/s11671-018-2775-z)
Supplement: Supplementary file 1 — Viability t- statistics of tumour cells. (DOCX 36 kb) [file 11671_2018_2775_MOESM1_ESM.docx]

**ADDITIONAL FILES**

**Tiny rare-earth fluoride nanoparticles activate tumor cell growth via electrical polar interactions**

V. V. Semashko, M. S. Pudovkin, A. C. Cefalas, P. V. Zelenikhin, V. E. Gavriil, A. S. Nizamutdinov, Z. Kollia, A. Ferraro and E. Sarantopoulou.

**Additional File 1 Viability t- statistics of tumor cells**

A t-test statistical analysis (p and Fisher F values) of tumour cells viabilities showed that the growth of tumour cells was un-saturated at 24 h; it followed an unknown physical law connecting viability and concentration of RE-NPs.

|  | **Cell Line** | **N** | **Mean growth** | **STD** | **F-test** | **p-value** |
| --- | --- | --- | --- | --- | --- | --- |
| **PrF_3_** | **A 549** | **6** | **141** | **10** | **56** | **0.01** |
|  |  | **6** | **110** | **5** |  |  |
|  |  | **6** | **104** | **1** |  |  |
|  | **SW 837** | **6** | **147** | **24** | **1.59** | **0.23** |
|  |  | **6** | **129** | **22** |  |  |
|  |  | **6** | **126** | **20** |  |  |
|  | **MCF 7** | **6** | **123** | **17** | **4.41** | **0.03** |
|  |  | **6** | **100** | **18** |  |  |
|  |  | **6** | **102** | **7** |  |  |
| **LaF_3_** | **A 549** | **6** | **125** | **24** | **1.56** | **0.24** |
|  |  | **6** | **115** | **4** |  |  |
|  |  | **6** | **104** | **3** |  |  |
|  | **SW 837** | **6** | **186** | **18** | **17.58** | **0.01** |
|  |  | **6** | **135** | **23** |  |  |
|  |  | **6** | **119** | **20** |  |  |
|  | **MCF 7** | **6** | **117** | **11** | **10.16** | **0.01** |
|  |  | **6** | **125** | **7** |  |  |
|  |  | **6** | **99** | **12** |  |  |
