# Supplementary material for: Tiny Rare-Earth Fluoride Nanoparticles Activate Tumour Cell Growth via Electrical Polar Interactions
Source: Nanoscale Res Lett. 2018 Nov 21;13:370. doi: 10.1186/s11671-018-2775-z (PMC6249154; doi:10.1186/s11671-018-2775-z)

**Additional File 2 NPs size t-statistics**

**.** Mean size of NPs, total errors and t-statistic test of RE-NPs in DMEM+FBS suspensions (0.1 Kgm^-3^) extracted from AFM, DLS, TEM and XRD data. Comparison of DLS with the TEM data indicated the formation of core-shell RE-NPs in the suspensions. For the statistics of the data and the level of confidence of mean values and errors, the t-statistic test was used with the “null hypothesis” that “the mean values, extracted from different random samples (images, digitized data) were equal with the mean value of each individual sample”. From the AFM images, only the mean equal area cycle (MEAC) diameter of PrF_3_ was accepted (p=0.001). The p-values of the mean size of MEAC diameters of NPs extracted from TEM and XRD data were 0.29 and 0.06 for PrF_3_ and LaF_3_, respectively. The data in the red cycle indicated the most reliable MEAC diameter values (p≤0.001).


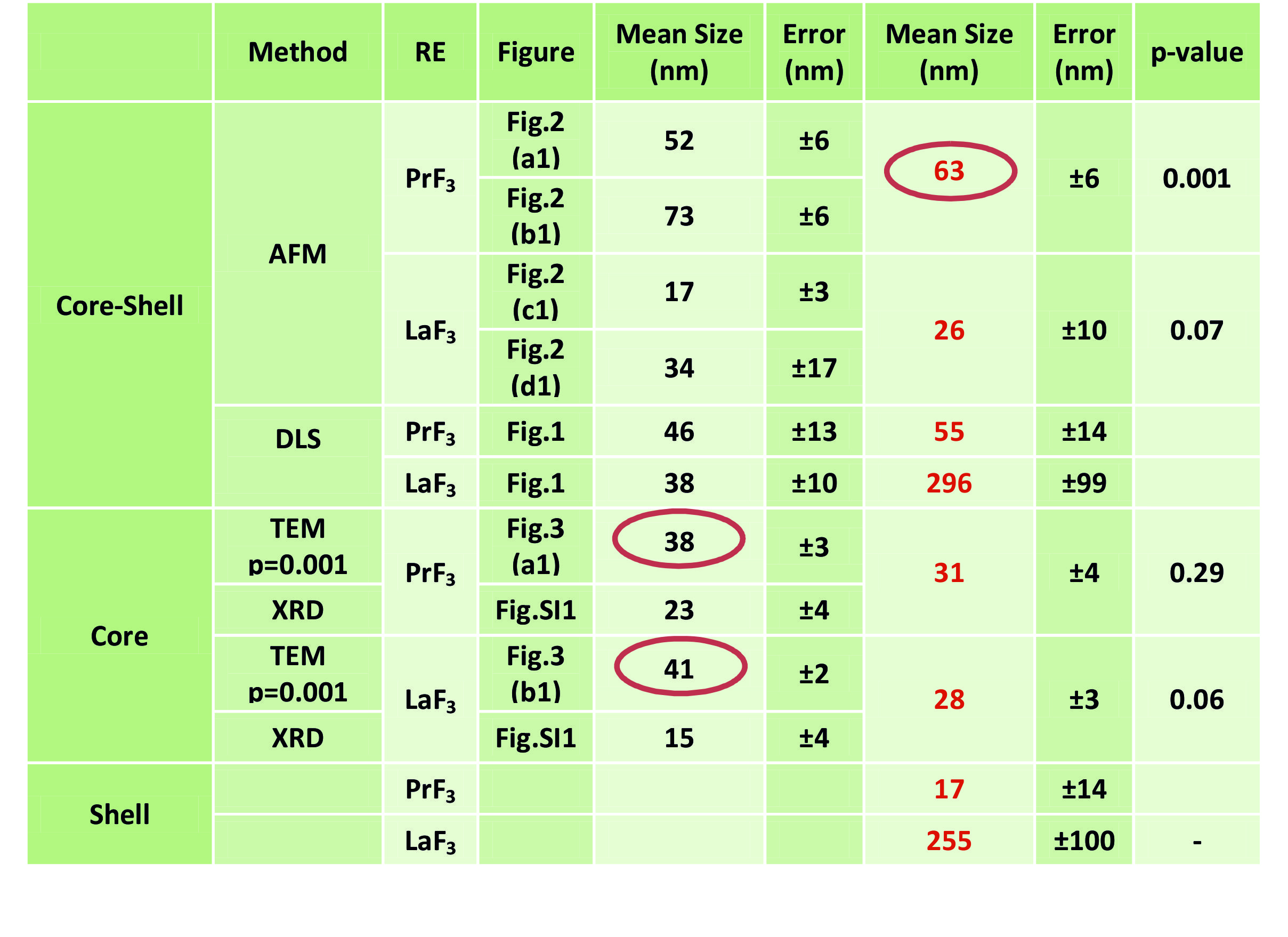

Supplement: Supplementary file 2 — NPs size t-statistics. (DOCX 2685 kb) [file 11671_2018_2775_MOESM2_ESM.docx]
